# Gut dysbiosis impairs hippocampal plasticity and behaviors by remodeling serum metabolome

**DOI:** 10.1080/19490976.2022.2104089

**Published:** 2022-07-24

**Authors:** Guoqiang Liu, Quntao Yu, Bo Tan, Xiao Ke, Chen Zhang, Hao Li, Tongmei Zhang, Youming Lu

**Affiliations:** a Department of Physiology, School of Basic Medicine and Tongji Medical College, Huazhong University of Science and Technology, Wuhan, province, China; b Wuhan Center of Brain Science, Huazhong University of Science and Technology, Wuhan, province, China; c Department of Pathophysiology, School of Basic Medicine and Tongji Medical College, Huazhong University of Science and Technology, Wuhan, province, China

**Keywords:** Gut microbiota, behavior, psychiatric disorders, serum metabolites, 4-methylphenol

## Abstract

Accumulating evidence suggests that gut microbiota as a critical mediator of gut-brain axis plays an important role in human health. Altered gut microbial profiles have been implicated in increasing the vulnerability of psychiatric disorders, such as autism, depression, and schizophrenia. However, the cellular and molecular mechanisms underlying the association remain unknown. Here, we modified the gut microbiome with antibiotics in newborn mice, and found that gut microbial alteration induced behavioral impairment by decreasing adult neurogenesis and long-term potentiation of synaptic transmission, and altering the gene expression profile in hippocampus. Reconstitution with normal gut flora produced therapeutic effects against both adult neurogenesis and behavioral deficits in the dysbiosis mice. Furthermore, our results show that circulating metabolites changes mediate the effect of gut dysbiosis on hippocampal plasticity and behavior outcomes. Elevating the serum 4-methylphenol, a small aromatic metabolite produced by gut bacteria, was found to induce autism spectrum disorder (ASD)-like behavior impairment and hippocampal dysfunction. Together our finding demonstrates that early-life gut dysbiosis and its correlated metabolites change contribute to hippocampal dysfunction and behavior impairment, hence highlight the potential microbiome-mediated therapies for treating psychiatric disorders.

## Introduction

The high comorbidity between psychiatric disorders and gastrointestinal diseases unveils an unexpected link between the gut and brain. Increasing evidence shows that gut microbiome is a critical regulator of gut-brain bidirectional interaction.^1^ Microbiota homeostasis has been linked to neurodevelopment and brain function, especially the behavior and emotional processing, such as the anxiety- and depressive-like behaviors,^2^ stress-related responsiveness,^3^ and fear-related behaviors.^4^ Notably, microbiota-derived metabolites and co-metabolites are known to be important mediators of the microbiome-center nervous system (CNS) signaling, including short-chain fatty acids, serotonin, and tryptophan-derived compounds.^5^ Currently, alterations in the gut microbiota composition have been implicated in several mental disorders, such as Alzheimer’s disease, autism, depression, and schizophrenia.^6^ However, whether gut dysbiosis plays a key role in the onset and exacerbation of such diseases remain unknown.

The neurodevelopment at the postnatal stage parallels with the establishment and maturation of the microbiome,^7^ therefore disturbance of the developing gut microbiota has the potential to significantly influence neurodevelopment and enhance the vulnerability of mental disorders. Previous studies have shown that maternal diet, stress, and infection are associated with autism, anxiety, and schizophrenia;^8^ an ancillary cohort study suggests that infant gut microbiome composition has a significant correlation with social and fine motor skills.^9^ Growing evidence based on germ-free (GF) mice suggests that complete absence of gut bacteria decreases the integrity of the blood-brain barrier,^10^ reduces the anxiety- and depression-like behaviors,^11^ induces social cognition impairment,^12^ leads to molecular changes in the prefrontal cortex,^13^ hippocampus,^14^ and amygdala.^4^ The hippocampus, a key brain area for learning, memory, and mood regulation, is particularly vulnerable to both internal and external stimuli, including the gut dysbiosis.^15^ Clinical studies have demonstrated that several psychiatric and neurodegenerative disorders are accompanied by impairment of hippocampal function.^16^ Emerging research has suggested that gut microbiota is implicated in adult neurogenesis^17^ and hippocampal plasticity.^18^ For example, GF mice showed increased hippocampal volume (10%) and adult hippocampal neurogenesis,^17^ and exhibited an alteration in hippocampal mRNA expression.^18,19^ Although GF mouse studies have provided critical insights into the role of gut microbiome in modulating CNS functions, they show limited translational equivalent, given the aseptic feeding conditions and immature immune system of GF mouse. Therefore, it is urgently needed to establish direct causality between early-life dysbiosis and brain disorders by introducing real-world insults, and then reveal the cellular and molecular mechanisms mediating the association.

Antibiotic interventions may provide us such a choice by the fact that antibiotics are the most prescribed medication to infants and children^20^ and are considered to be the most important cause of early-life dysbiosis in humans. Emerging epidemiological studies have shown that gut microbiota changes following antibiotic exposure in the first year of life were associated with increasing the risk of allergies, asthma, obesity, and inferior cognitive.^21,22^ Furthermore, neonatal prebiotic intake was found to have long-term effects on hippocampal physiology, brain development, and behavior.^23^ The postnatal early-life is not only a critical window for neurodevelopmental vulnerability but also an optimal period for therapeutic interventions of gut microbiota-related brain disorders. Thus, future efforts should focus on revealing the biological basis of early-life microbiota–CNS interactions.

In this study, we generated a gut dysbiosis mouse model by using two antibiotics widely used in clinic, ampicillin (Amp) versus vancomycin (Van). Our study shows that disruption of the microbiome induced anxiety-like behaviors, impaired social novelty, and spatial memory. Behavior impairments were found to be closely associated with reducing adult neurogenesis, decreasing synaptic plasticity, altering gene expression in hippocampus and serum metabolites. Reconstitution with normal gut microbiota rescued the behavioral deficits. Finally, we found that increasing the blood level of bacterial metabolite 4-methylphenol (*p*-cresol) induced autism spectrum disorder (ASD)-like behavior impairment and hippocampal dysfunction.

## Results

### Antibiotic treatment disrupts the gut microbial composition

**Figure 1.**
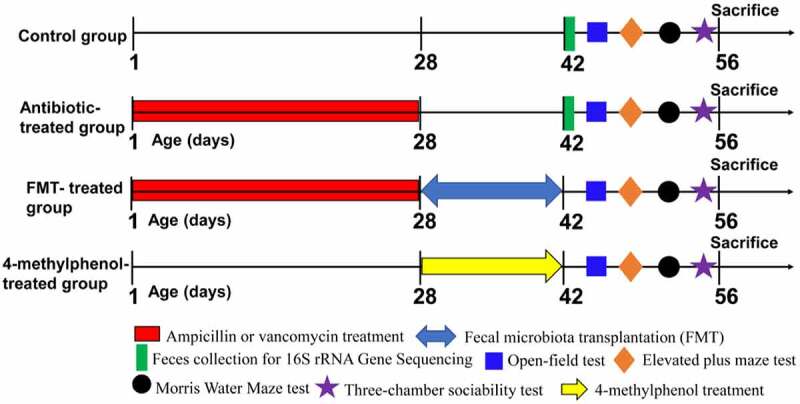
Experimental group and time linesThe newborn mice (P1) were treated with antibiotics (100 mg/kg ampicillin or 50 mg/kg vancomycin) or vehicle (distilled water) by oral gavage daily for four weeks (P1-P28). The feces of the mice were collected at the day P42 for 16S rRNA sequencing. Then, a series of behavioral testing was conducted in the order of OFT, EPM, MWM and three-chamber social test (P43-P56). For FMT-treated group, a fecal microbiota transfer protocol was conducted (P28-P42) in antibiotic-treated mice after the final antibiotic gavage. After that, animals were subjected to behavioral tests mentioned above (P43-P56). For 4-methylphenol-treated group, wild-type mice (4-week-old C57BL/6 J mice) were injected i.p. with 4-methylphenol (35 mg/kg) or saline (control group) daily for two weeks (P28-P42). Behavioral testing was performed after the treatment. After the social behavior test, the mice were returned to the their home cages for 1.5 h to induce the expression of IEGs, and then mice were perfused or the brain tissues were collected immediately.

**Figure 2.**
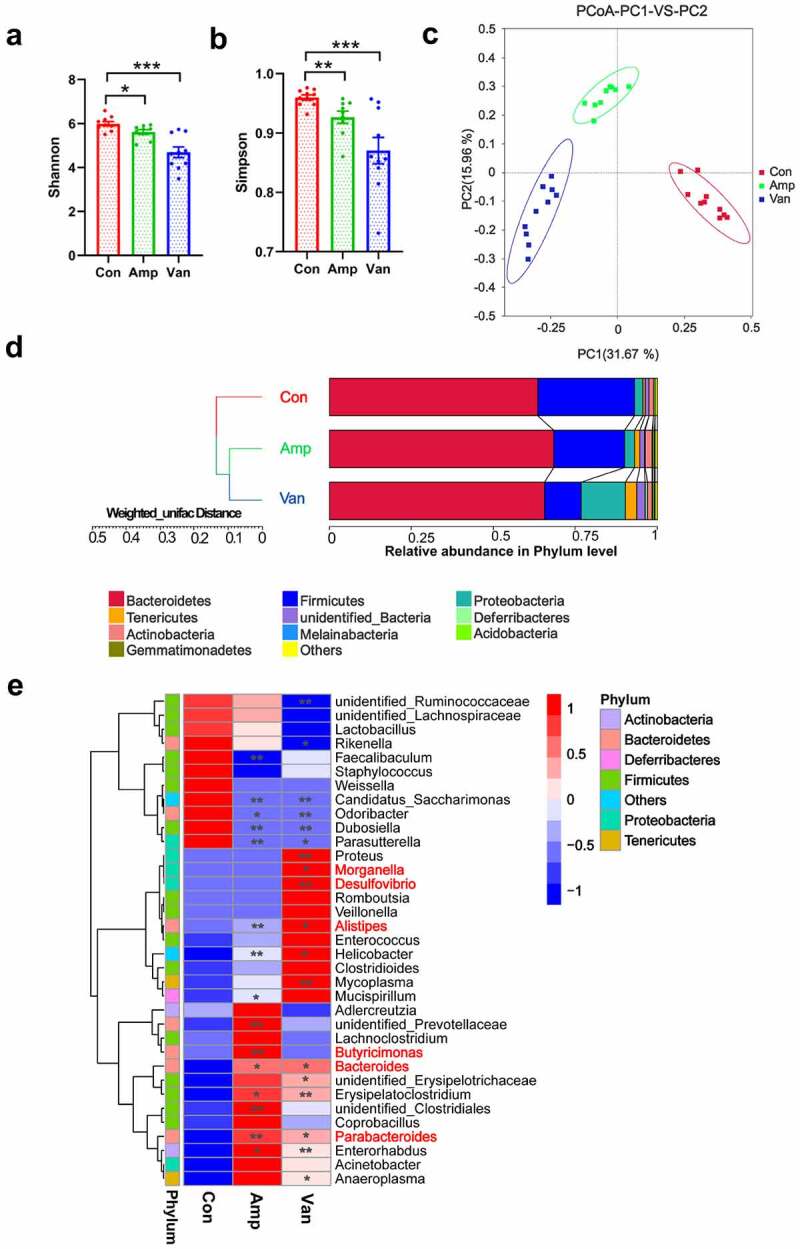
Antibiotic treatment induces dramatic changes in the gut microbial composition(a) Shannon index and (b) Simpson index of gut microbiota. Data are presented as mean ± SEM, N = 9–10/group, unpaired two-tailed *t*-test, *P < 0.05, **P < 0.01, ***P < 0.001. (c) PCoA analysis based on the Bray-Curtis distance was performed to visually explore the similarity and variations between the samples’ microbial composition. N = 9–10/group. The percentages in parentheses refer to the proportions of variation explained by each ordination axis. (d) UPGMA clustering tree based on weighted UniFrac distance shows the relative abundance of gut microbiota at the phylum level. N = 9–10/group. (e) Heatmap of the relative abundance of top 35 bacterial genera. Some bacteria associated with neurological and psychiatric disorders were marked in red. N = 9–10/group. Data are presented as mean ± SEM, unpaired two-tailed *t*-test, *P < 0.05, **P < 0.01.

We created a dysbiosis mouse model by treating newborn mice with Amp or Van for four weeks (P1-P28)(Figure 1). Two weeks after the final antibiotic treatment, the feces of the mice were collected for 16S rDNA high-throughput sequencing. As expected, neonatal antibiotic exposure induced dramatic changes in the gut microbial composition. Mice exposed to antibiotic displayed lower alpha diversity (species diversity) as calculated by Shannon and Simpson indices Figure 2(a,b). Principal Coordinates Analysis (PCoA) based on the Bray-Curtis distance revealed a clear difference in bacteria structure between the vehicle-treated group (Con) and antibiotic-treated group (Amp and Van) Figure 2c. At the phylum level, antibiotic-treated mice featured a decrease of *Firmicutes* and an increase of *proteobacteria* Figure 2d. Better separation of vehicle-treated and antibiotic-treated mice was observed at the genus level. The abundance of 35 dominating genera significantly changed following antibiotic intervention Figure 2e. Among them, some bacteria were associated with neurological and psychiatric disorders (FigureS1). For example, the *Alistipes, Megamonas*, and *Parabacteroides* were reported to be elevated in patients with anxiety and depression;^24^ the *Alistipes* and *Butyricimonas* were found to be negatively correlated with cognition ability;^25^ the *Desulfovibrio, Parabacteroides*, and *Bacteroides* increased significantly in autism patients and autism model mice.^26,27^

## Gut dysbiosis during early-life induces anxiety and impairs spatial memory

**Figure 3.**
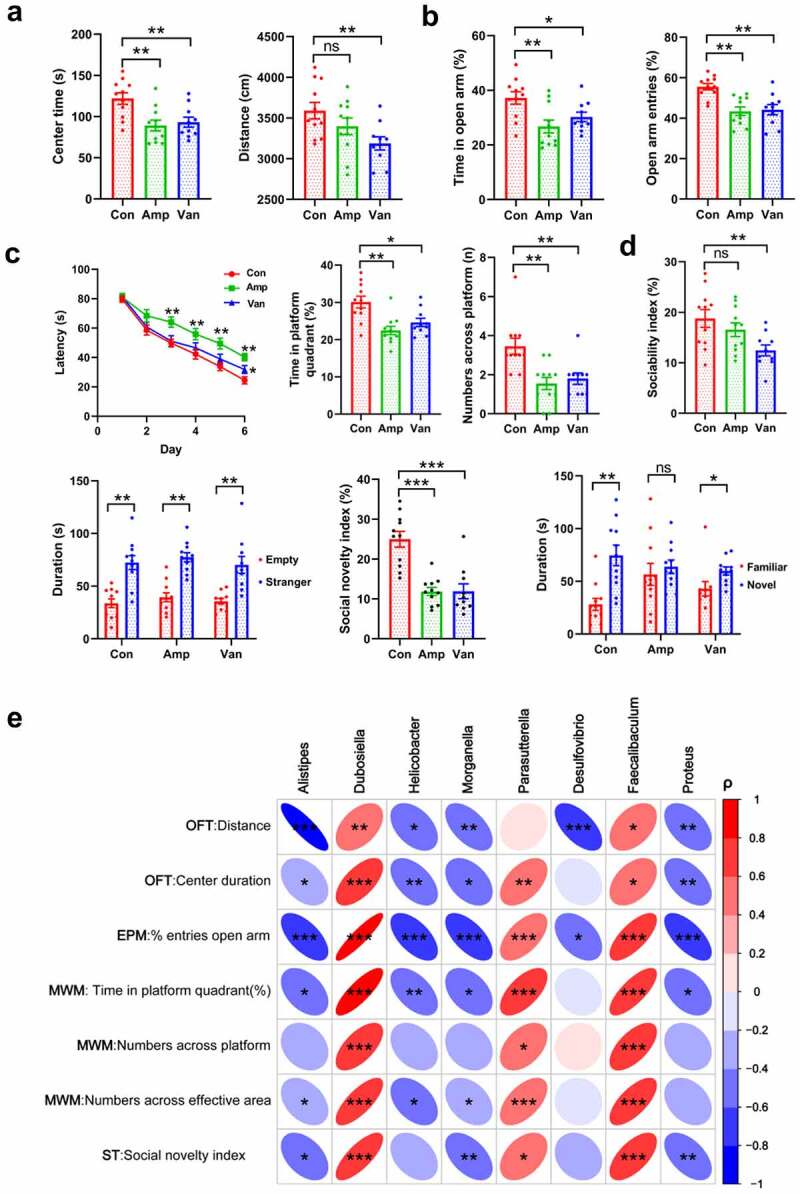
Gut dysbiosis increases the anxiety and impairs the spatial memory and social behavior(a, b) The locomotor and anxiety-like behavior in the OFT (A) and EPM test (B). N = 9–10/group. Data are presented as mean ± SEM, unpaired two-tailed *t*-test, *P < 0.05, **P < 0.01, ns: no significance. (c) The spatial learning and memory ability were evaluated using the MWM test. N = 9–10/group. Data are presented as mean ± SEM, unpaired two-tailed *t*-test, *P < 0.05, **P < 0.01. (d) The figure shows the three-chambered social test, the sociability index and social novelty index were shown. N = 9–10/group. Data are presented as mean ± SEM, unpaired two-tailed *t*-test, **P < 0.01, ***P < 0.001, ns: no significance. (e) Spearman’s correlation analysis of the relationship between behavioral outcomes and the abundance of bacteria. Positive sloped elipses represents a positive correlation, negatively sloped ellipses indicates negative correlations. The color shows the strength of the spearman correlation coefficient ρ, ρ > 0.6 or ρ<-0.6, and *P*-value < 0.05 were considered as a significant correlation. *P < 0.05, **P < 0.01, ***P < 0.001.

We conducted a series of behavioral tests to determine whether gut dysbiosis in early life is sufficient to lead to any changes in phenotype development. Our data suggest that antibiotic-treated mice significantly reduced the total traveling distance and the time spent in the center arena in the open-field test (OFT) compared with the vehicle-treated control Figure 3a, indicating a decrease in locomotor activity and an increase in anxiety-like behavior. Likewise, the elevated plus maze (EPM) test, another anxiety-related behavioral test, shows that antibiotic-treated mice spent less time traveling in the open arm and decreased the number of open arm entries Figure 3b. Spatial learning and memory were assessed using the Morris water maze (MWM) test. We found that Amp-treated mice had longer escape latencies from training day 3 to day 6 compared with vehicle-treated control, and similar performance was found in Van-treated mice on day 6 Figure 3c. The probe trial results demonstrate that antibiotic-treated mice remarkably reduced the time traveling in the target quadrant and the number of times across the platform area, showing obvious spatial memory impairment Figure 3c. In the three-chamber social test, Amp-treated mice showed an impairment in social novelty, while the sociability was found to be normal compared with the control group. The Van-treated mice exhibited a decreased sociability and a deficit in social novelty Figure 3d. Taken together, our results reveal that disruption of gut microbiota in early-life induced anxiety-like behavior and impaired social novelty and spatial memory.

We next analyzed whether these behavioral outcomes co-vary with specific bacteria and found that the abundance of eight bacterial genera significantly correlated with several behavioral indexes by using Spearman’s correlation analysis Figure 3e. The high abundance of *Alistipes, Helicobacter, Morganella, Desulfovibrio*, and *Proteus* negatively correlated with the locomotion activity and open arm entries in antibiotic-treated mice. While the *Dubosiella, Parasutterella*, and *Faecalibaculum* showed the opposite effects, as they were not only positively correlated with increasing the center duration in the OFT and open arm entries in the EPM test but also showed positive correlations with improving the performance in the MWM test and three-chamber social test. It is worth noting that alteration of microbial communities mentioned above has been described in patients with psychiatric disorders, including the anxiety, depression, autism, and cognitive dysfunction.^24–27^ Together, these associations between specific bacterial genera and behavioral outcomes provided further evidence that specific bacteria may contribute to modulate behavior.

## Disruption of gut microbiota impairs synaptic plasticity and adult neurogenesis

**Figure 4.**
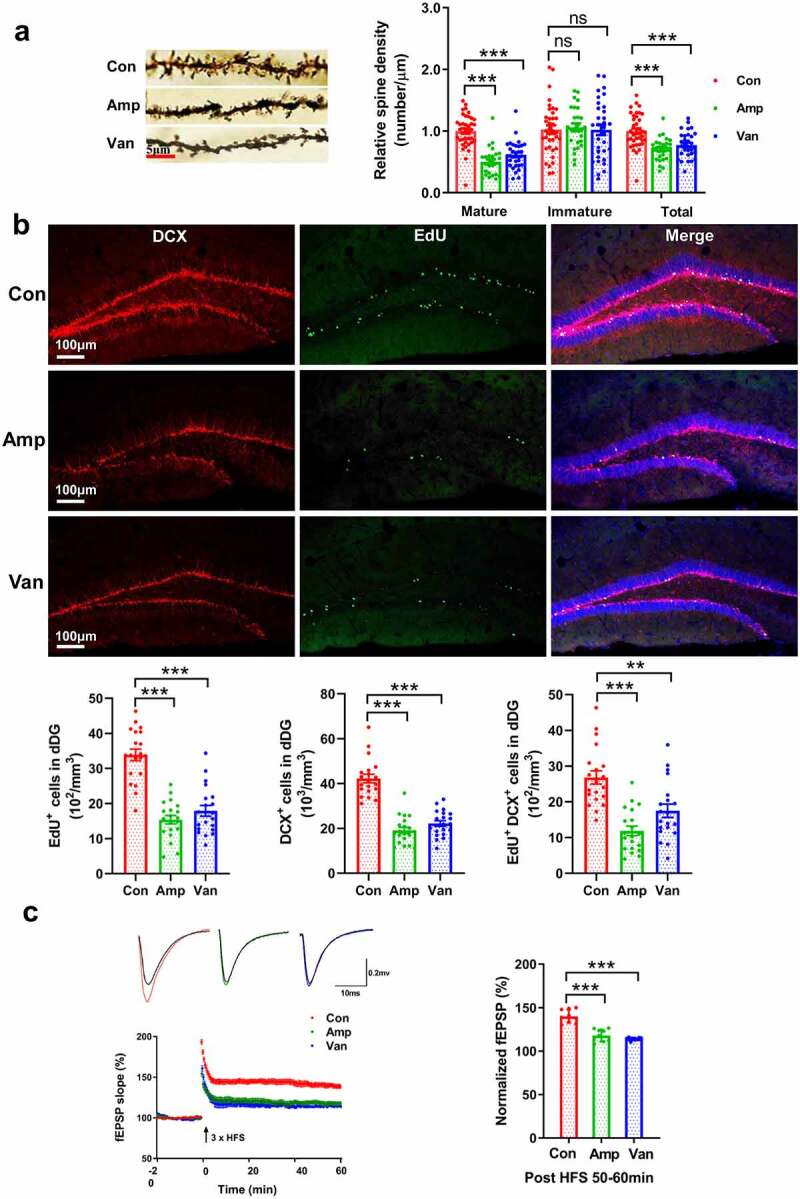
Disruption of gut microbiota impairs synaptic plasticity and adult neurogenesis(a) Golgi staining was used to visualize apical dendritic processes in the dorsal DG (dDG), the mature-appearing spine (mushroom spine) decreased following disruption of gut microbiota. N = 3/group. Data are presented as mean ± SEM, unpaired two-tailed *t*-test, **P < 0.01, ***P < 0.001, ns: no significance. (b) The representative micrographs and quantitative analysis of the cells positive for EdU and DCX in the dorsal hippocampus. N = 3/group. Data are presented as mean ± SEM, unpaired two-tailed *t*-test, **P < 0.01, ***P < 0.001. (c) Gut dysbiosis impaired LTP in antibiotic-treated mice, The time course and extent of LTP induction following HFS were shown. N = 9/group. Data are presented as mean ± SEM, unpaired two-tailed *t*-test, ***P < 0.001.

The hippocampus, a key brain region involved in various sensory, emotional, and cognitive function, is highly susceptible to various damage.^16^ Previous studies have shown that the absence of gut microbiota affects the structure and function of the hippocampus.^17,18^ Therefore, we first analyzed whether early-life dysbiosis has an effect on dendritic spine morphogenesis. Compared with the control group, antibiotic-treated mice exhibited fewer apical dendritic spines with mature-appearing morphology (mushroom spine) in the dentate gyrus (DG) of the dorsal hippocampus Figure 4a and CA1 of the ventral hippocampus (FigureS2A), suggesting that a healthy early-life gut microbiota promotes spine maturation. Dendritic spines are highly dynamic neural structures, alterations in their morphology have been recognized as critical for synaptic plasticity and long-term memory.^28^ Next, we determined the effect of gut dysbiosis on hippocampal neurogenesis, which has been considered as a cellular model of anxiety^29^ and spatial memory.^30^ We used antibodies against 5-ethynyl-2’-deoxyuridine (EdU) and doublecortin (DCX) to label proliferation and neuronal differentiation of adult hippocampal progenitor cells. The number of cells positive for EdU, DCX, or double-labeled DCX-EdU cells decreases significantly after antibiotic intervention, revealing an impaired adult neurogenesis in antibiotic-treated mice compared with the controls (Figure 4b, FigureS2B).

Subsequently, we evaluated whether disruption of gut microbiota exerted any effect on long-term potentiation (LTP) of synaptic transmission, the main form of synaptic plasticity, as a cellular substrate of learning and memory.^31^ The field excitatory postsynaptic potentials (fEPSPs) were recorded. Application of a brief high-frequency stimulation (tetanus) induced LTP of fEPSP in vehicle-treated group, whereas only transient increase of fEPSP was observed in antibiotic-treated mice Figure 4c, indicating an impaired LTP.

## Disruption of gut microbiota alters the gene expression in the hippocampus

**Figure 5.**
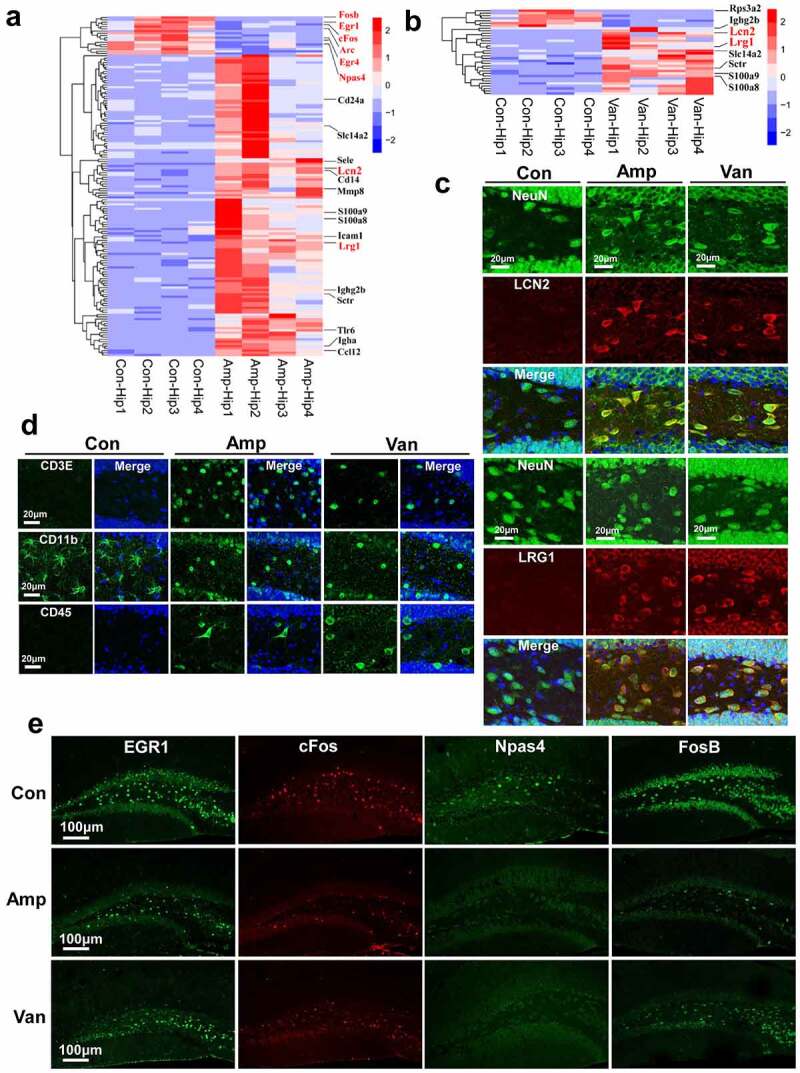
Disruption of gut microbiota alters hippocampal gene expression(a-b) Heatmap of significantly differential genes in the hippocampus following disruption of gut microbiota, some representative genes were marked in red in the graph. N = 4/group, FDR adjusted *P*-value of 0.1 and absolute foldchange of 2 was set as the threshold for significantly differential expression. (c) Confocal imaging shows that the LCN2- and LRG1-positive cells significantly increased in hippocampal DG following disruption of gut microbiota. N = 5/group. The nuclei were stained with DAPI. (d) The representative micrographs show the CD3E-, CD11b- and CD45-positive cell in the hippocampal DG. N = 5/group. The nuclei were stained with DAPI. (e) The representative micrographs show that antibiotic treatment resulted in an impairment the up-regulation of IEGs induced by social behavior test. N = 5/group.

To identify the molecular determinants underlying the differences in behavioral outcomes in antibiotic-treated mice, we performed an unbiased transcriptomic analysis of the hippocampus. We found significant changes in the expression levels of 149 genes and 34 genes in Amp-treated and Van-treated mice (FDR<0.1, fold change>2), compared with the wild-type control Figure (5a,b). Eleven common differential genes shared by them mainly encode proteins related to immunity and inflammation, including immunoglobulin heavy constant gamma 2B gene (*Ighg2b*), lipocalin-2 (*Lcn2*), and S100 calcium-binding protein A8 and A9 coding genes (*S100a8, S100a9*) (FigureS3A). Of particular interest is the expression of LCN2 and leucine-rich α2-glycoprotein (LRG1) significantly increased in hippocampal neurons (Figure 5c, FigureS3B). While overexpression of LCN2 in hippocampal neurons has been reported to decrease the spine density and inhibit spine maturation.^32^ Hippocampal LRG1 overexpression has been shown to impair memory by inducing synaptic dysfunction and decreasing excitatory postsynaptic potentials.^33^

Functional enrichment analysis suggests that most of the up-regulated genes fall into the broad categories of immune response and process, and apoptotic process (FigureS3C). We found that gut dysbiosis increased the number of cleaved caspase-3 positive neuron (FigureS3D) and induced neuron loss in the hippocampus (FigureS3E). Transcriptional data show that disruption of gut microbiota increased the expression of pathogens recognition genes (*Cd14, Tlr6*), immunoglobulin genes (*Ighg2b, Igha*), inflammatory response genes (*Ccl12, S100a8*, and *S100a9*), and leukocyte recruitment genes (*Sele, Icam1* and *Cd24a*). We also found that the number of CD3E-, CD11b- and CD45-positive cell remarkably increased in the hippocampus of antibiotic-treated mice Figure 5d. This result is consistent with the previous reports that gut dysbiosis may stimulate the immune response and promote migration and infiltration of the immune cell into the brain.^34^ Additionally, we observed that the protein expression of immediate early genes (IEGs), including the *Egr1, FosB, Npas4*, and *cFos*, were highly induced after the social behavior test in the hippocampal DG of the wild-type mice, while antibiotic treatment resulted in an impairment in the protein expression of IEGs (Figure 5e, FigureS3B, and FigureS4). IEGs predominantly encode early-response transcription factors, which have been reported to be induced by a wide range of stimuli, such as novel objects and social stress, and they have been implicated in numerous neuronal functions such as synaptic plasticity, learning, memory, and spatial exploration.^35^

## Reconstitution with normal gut microbiota rescues the behavioral deficits in antibiotic-treated mice

**Figure 6.**
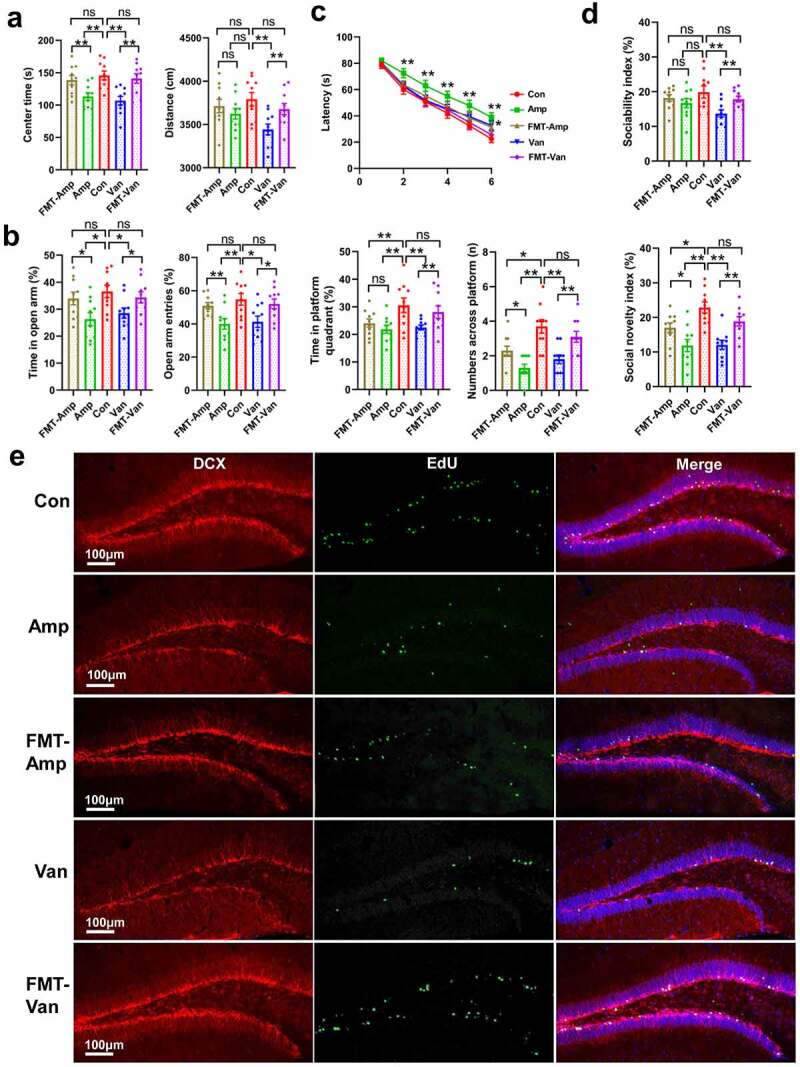
Reconstitution with normal gut flora produces the therapeutic effects in the gut dysbiosis mice(a-b) FMT restored the locomotor ability and removed the anxiety-like behavior in gut dysbiosis mice. (a) OFT, (b) EPM test. N = 9–10/group. Data are presented as mean ± SEM, unpaired two-tailed *t*-test, *P < 0.05, **P < 0.01, ns: no significance. (c-d) FMT rescued the deficits in spatial memory (c) and social activity (D) in the gut dysbiosis mice. (c) MWM test; (d) three-chamber social test. N = 9–10/group. Data are presented as mean ± SEM, unpaired two-tailed *t*-test, *P < 0.05, **P < 0.01, ns: no significance.(e) Confocal micrographs show that FMT rescued the adult neurogenesis in antibiotic-treated mice. N = 5/group.

Having shown that gut dysbiosis-induced behavior deficits and altered the gene expression profiles in the hippocampus, therefore we investigated whether reconstitution with normal gut flora produces therapeutic effects in gut dysbiosis mice. Remarkably, we found that behavior deficits were significantly ameliorated in antibiotic-treated mice by transplanting the intestinal microbiota from healthy donor (the same age SPF mice) Figure 6(a,d). The fecal microbiota transplantation (FMT)-treated mice did not exhibit anxiety-like behavior, as shown by restoring the duration of time spent in the center zone in the OFT Figure 6a and the number of open arm entries in the EPM test Figure 6b. FMT significantly improved the ability of spatial learning and memory and preference for social novelty in Amp-treated mice, as indicated by remarkably increasing the time-traveling in the target quadrant and the number of times across the platform in MWM test Figure 6c, and extending the contact mean duration in three-chamber social test Figure 6d, FigureS5A). While Van-treated mice transplanted with a normal microbiota nearly restored all parameters to control levels in the MWM test Figure 6c and three-chamber social test (Figure 6d, FigureS5A).

**Figure 7.**
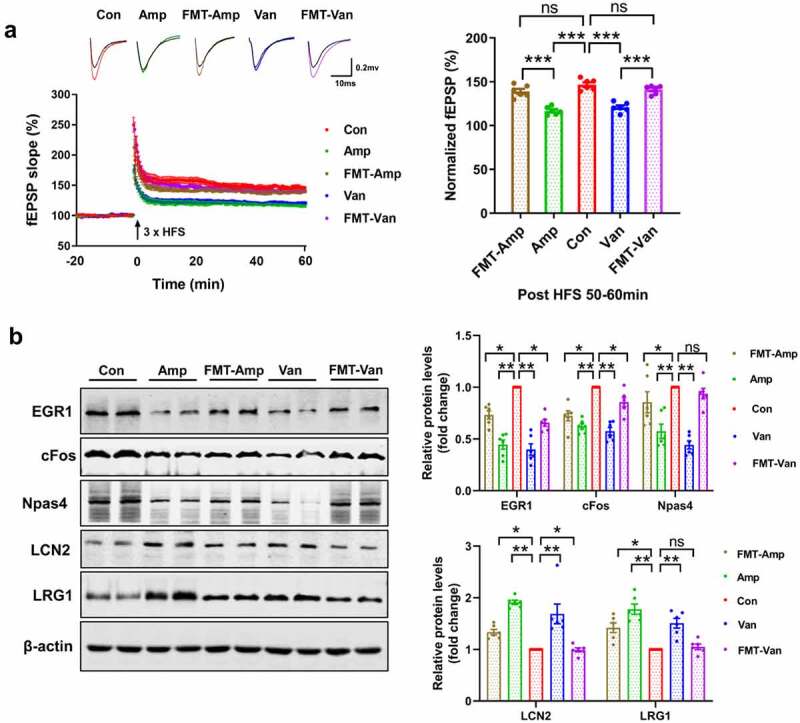
FMT restore the hippocampal LTP and the expression of IEGs (a) FMT restored the hippocampal LTP in the gut dysbiosis mice. The time course and extent of LTP induction following HFS were shown. N = 6/group. Data are presented as mean ± SEM, unpaired two-tailed *t*-test, ***P < 0.001. (b) FMT rescued the expression of IEGs, LCN2 and LRG1. N = 6/group. Data are presented as mean ± SEM, unpaired two-tailed *t*-test, *P < 0.05, **P < 0.01, ns: no significance.

Importantly, we found that FMT not only rescued the behavior deficits but also restored the adult neurogenesis (Figure 6e, FigureS5B, and FigureS6) and hippocampal LTP Figure 7a in antibiotic-treated mice. Our results also show that reconstitution with normal microbiota rescued the expression of IEGs, LRG1, and LCN2 Figure 7b. Meanwhile, the hippocampal mRNA levels of pathogens recognition gene (*Tlr6*), leukocyte recruitment genes (*Icam1* and *Lsp1*) and proinflammatory gene (*Ccl12*) were also restored in the antibiotic-treated mice following the FMT (FigureS5C). Taken together, the above results indicate that disruption of the microbiome in early-life plays a causal role for the behavioral disorders, transcriptional changes in the hippocampus and adult neurogenesis impairment, highlighting the importance of gut microbiota in modulating brain development and behavior.

## Alteration in the gut microbiota induces changes of serum metabolites

**Figure 8.**
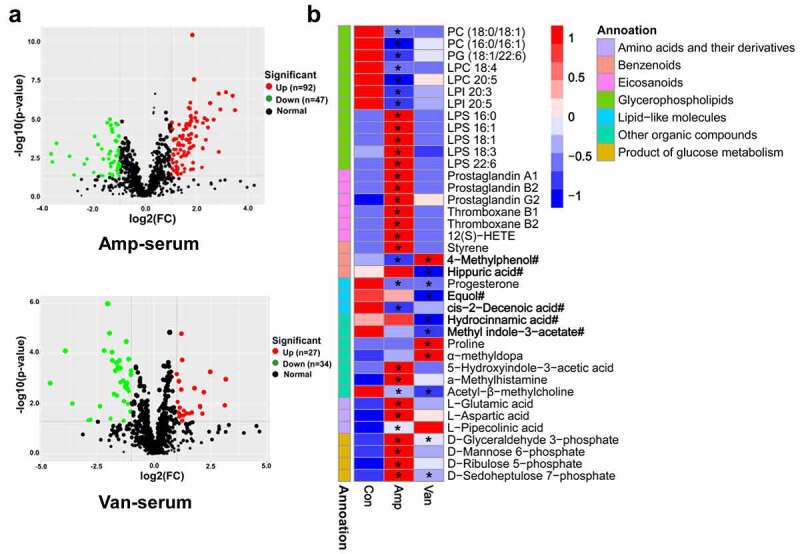
Microbiota dysbiosis in early-life alters serum metabolome(a) volcano plot showing differential metabolites in amp-treated and van-treated mice. N = 9–10/group. Criteria for significant differences (VIP > 1, *P* < 0.05 and fold change ≥ 2). (b) heatmap of representative differential metabolites. The metabolites derived from the gut bacteria were marked with “**#**”. *P < 0.05, **P < 0.01.

More and more studies have shown that gut microbiota is not only involved in nutrient absorption and energy metabolism,^36^ but also participate in modulating brain development by producing bioactive metabolites, such as short-chain fatty acids and neurotransmitters.^37^ We utilized a UHPLC-MS/MS-based untargeted metabolomics approach to determine whether the serum metabolome is affected by disruption of gut microbiota. Overall, 946 known metabolites were detected in positive and negative ion modes from the serum. Compared with the vehicle-treated controls, 139 metabolites were found to be significantly changed (VIP > 1 and *P*-value < 0.05 and fold change > 2) in the serum of Amp-treated mice, while only 60 metabolites remarkably changed in Van-treated mice Figure 8a, and 27 metabolites are shared by these two groups (FigureS7A).

Functionally, the differential metabolites are mainly involved in lipid, benzenoids, amino acid, carbohydrate, and organic acids metabolism (Figure 8b, FigureS7B). Interestingly, lipid metabolites were mainly enriched in two categories, and many of them are highly permeable to the blood-brain barrier, and may have effects on neuroinflammation and neurological function, such as eicosanoids derivatives, glycerophospholipids. The prostaglandin and thromboxane, pro-inflammatory metabolites of eicosanoids, were reported to be involved in numerous pathophysiological pathways;^38^ 12(S)-HETE, an eicosanoids derivative reported to be upregulated in patients with Alzheimer’s disease, was shown to be involved in modulating the metabolism of Aβ and tau, synaptic integrity.^39^

In addition to lipid metabolites, several molecules are also potentially associated with neurological dysfunction (Figure 8b). Among them, excessive α-methyldopa was reported to depress spontaneous locomotor activity in mice;^40^ a high level of proline reduced creatine kinase activity and induced oxidative stress in rat brain;^41^ progesterone, a gonadal steroid hormone, exerts neuroprotective/modulatory effects through its systemic anti-inflammatory and BDNF regulatory actions.^42^ Furthermore, we found that several remarkably shifted metabolites are derived from gut microbiota Figure 8b, including 4-methylphenol, equol, 5-hydroxyindole-3-Acetic Acid, and hippuric acid.^43^ Of particular interest is the metabolite 4-methylphenol, a toxic aromatic compound produced in the gut by anaerobic bacteria belonged to the *Coriobacteriaceae* and *Clostridium* fermenting tyrosine or toluene.^44^ 4-methylphenol has been proposed as a possible urine biomarker for autism, and it was detected at high levels in the serum of offspring of maternal immune activation (MIA)-treated mice.^45^ Together, our results reveal that alteration in the gut microbiota in early-life were sufficient to cause significant changes in serum metabolome, and metabolites changes may contribute to abnormal brain development and behavior.

## Serum metabolite 4-methylphenol impairs hippocampal plasticity and social behavior

**Figure 9.**
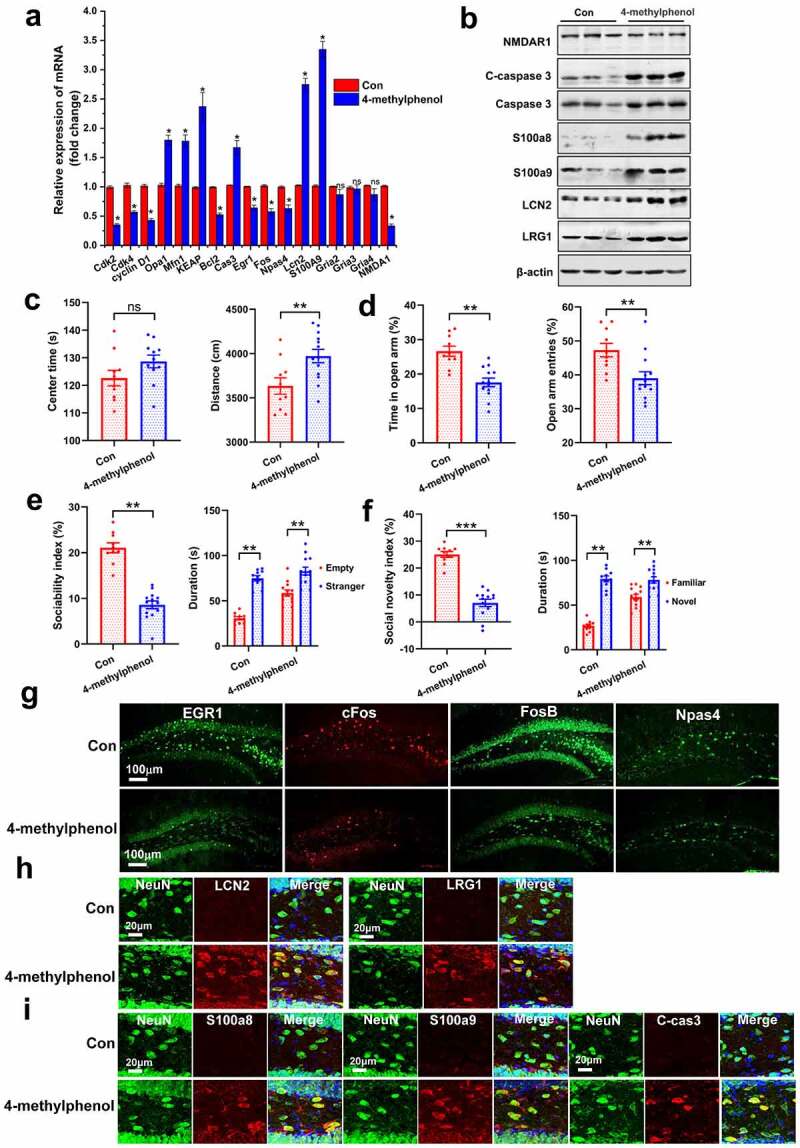
Bacterial metabolite 4-methylphenol impairs the social behavior and hippocampal plasticity(a) real-time RT-PCR and (b) western blot analysis show the differential genes in primary hippocampal neurons exposed to 4-methylphenol. Cells were treated with 4-methylphenol at 60 μM for 48 hours as well as DMSO as a control. Data are presented as mean ± SEM, unpaired two-tailed *t*-test, *P < 0.05, ns: no significance. (c-d) effects of 4-methylphenol on locomotor activity and anxiety-like behavior in the OFT (c) and EPM test (d). N = 10/group. Data are presented as mean ± SEM, unpaired two-tailed *t*-test, **P < 0.01, ns: no significance. (e-f) administration of the 4-methylphenol to native mice impaired the sociability (e) and social novelty (f). wild-type mice (4-week old) were injected i.p. with saline or 4-methylphenol (35 mg/kg) daily for two weeks. N = 10/group. Data are presented as mean ± SEM, unpaired two-tailed *t*-test, **P < 0.01, ***P < 0.001. (g) representative micrographs show that 4-methylphenol treatment reduced the up-regulation expression of IEGs induced by social test. N = 4/group. (h) representative micrographs show that 4-methylphenol treatment increased the number of LCN2 and LRG1 positive neuron in the hippocampal DG. N = 4/group. (i) confocal micrographs show that the protein of S100a8, S100a9 and cleaved-caspase-3 were overexpressed in the hippocampus of mice treated with 4-methylphenol. N = 4/group.

As mentioned above, several metabolites derived from gut microbiota significantly changed in serum following disruption of microbiome. Do these molecules play a role in behavioral deficits relevant to gut dysbiosis? To test this hypothesis, the metabolite 4-methylphenol was selected for further verification. Previous studies reported that exposure to 4-methylphenol induced mitochondrial dysfunction in mesenchymal stem cells,^46^ so we first determined whether it has the same effect on primary hippocampal neurons. We found that 4-methylphenol exposure enhanced the expression of mitofusion-associated genes (*Mfn1* and *Opa1*) and pro-apoptosis gene caspase-3, decreased the cell-cycle-associated genes (*Cdk2, Cdk4*, and *cyclin D1*) and the anti-apoptosis gene *Bcl2* by real-time RT-PCR Figure 9a. In addition, 4-methylphenol exposure significantly decreased the expression of c*Fos, Egr1, Npas4*, and NMDAR1, and enhanced the expression of *Lcn2* and *S100a9*. Furthermore, western blot analysis revealed that several pro-inflammatory and pro-apoptotic proteins were also overexpressed after treatment with 4-methylphenol, such as S100a8, S100a9, cleaved-caspase-3 Figure 9b.

Next, we investigated whether increasing serum 4-methylphenol is sufficient to cause any behavioral abnormalities in naive mice. Mice (4-week old) were treated with 4-methylphenol or vehicle, daily for two weeks. Compared to vehicle-treated controls, 4-methylphenol-treated mice increase the locomotor activity in the OFT Figure 9c, display some anxiety-like behavior by reducing the number of open arm entries and the duration of time spent traveling in the open arm of the EPM test Figure 9d. Likewise, 4-methylphenol-treated mice exhibit social impairment, as indicated by decreasing the sociability index and social novelty index Figure 9(e,f). Furthermore, we found that 4-methylphenol treatment significantly reduced the up-regulation of IEGs expression induced by social behaviors test (Figure 9g, FigureS8A), and increased the expression of LCN2, LRG1, S100a8, S100a9 and cleaved-caspase 3 in the hippocampal cells Figure 9(h,i). We also observed that the hippocampal CA1 and DG neurons were lost with the enhancement of inflammatory and apoptotic signals (FigureS8B-S8C). Overexpression of LCN2 and LRG1 in hippocampus has been shown to inhibit spine maturation^32^ and induce synaptic dysfunction.^33^ The above data demonstrate the bacterial metabolite 4-methylphenol induced behavior impairment by decreasing hippocampal plasticity and promoting inflammation and apoptosis of neurons.

Taken together, our results suggest that elevating serum levels of 4-methylphenol specifically caused ASD-like behavioral impairment by increasing anxiety and reducing social activity, indicating that metabolomic changes in early-life contribute to the onset and development of psychiatric disorders. Given that so many serum metabolites remarkably changed following gut dysbacteriosis, more complex behavioral changes may be modulated by combinations of these metabolites.

## Associations of host serum metabolites with gut microbiota

**Figure 10.**
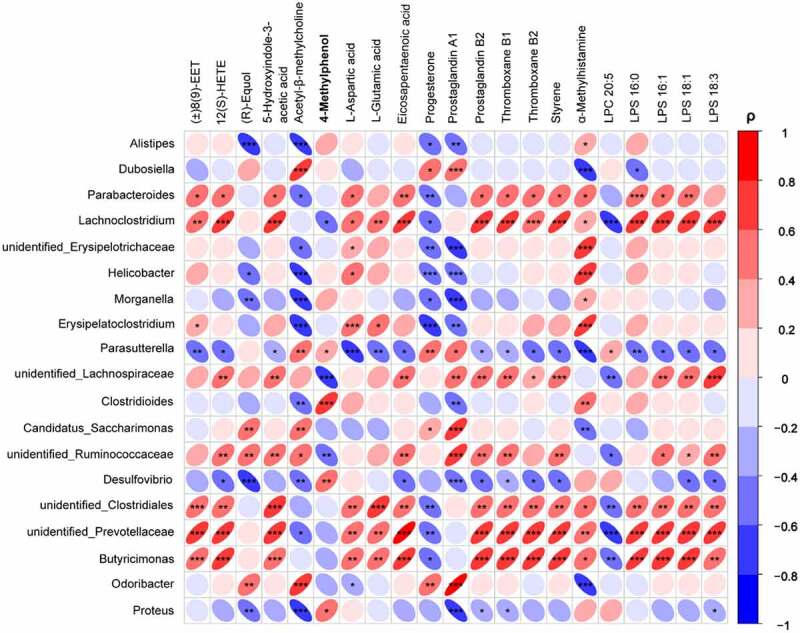
Heatmap analysis of the Spearman correlation between serum differential metabolites and dominanting bacteria genera.Negatively sloped ellipses indicates negative correlations, and positive sloped elipses represents a positive correlation. The color shows the strength of the spearman correlation coefficient ρ, ρ > 0.6 or ρ<-0.6, and *P*-value < 0.05 were considered as a significant correlation. *P < 0.05, **P < 0.01, ***P < 0.001.

To investigate the potential dependencies between altered gut microbiota and perturbed serum metabolites, Spearman correlation coefficient between differential metabolites and the abundance of gut bacteria were assessed and displayed in the form of a heat map (Figure 10). Our results show that the levels of many neuroactive metabolites were significantly correlated with the relative abundance of dominating microbial genera (coefficient (ρ) > 0.6, *P* < 0.05). The metabolite 4-methylphenol showed a significant positive correlation with the *Clostridioides*, and this result is consistent with previous reports that bacteria producing 4-methylphenol mainly belonged to *Clostridium* clusters.^44^ The proinflammatory metabolites proteinoids (prostaglandin B2, thromboxane B1, and B2) and eicosanoids derivatives ((±)8(9)-EET and 12(S)-HETE) were significantly positively correlated with the *Lachnoclostridium*, unidentified_*Prevotellaceae* and *Butyricimonas*, and a similar positive correlation was observed for the metabolites lysophosphatidylserines (LPS), eicosapentaenoic acid, L-aspartic acid, and L-glutamic acid. In contrast, the metabolites (R)-equol with neuroprotective effects exhibited a significantly negative correlation with the opportunistic pathogens *Alistipes*, and *Desulfovibrio* enriched in the antibiotic-treated mice. In addition, we find that some bacteria, including the *Lachnoclostridium, unidentified_Prevotellaceae* and *Butyricimonas*, may play critical roles in maintaining the metabolic homeostasis of the host by the fact that they are correlated with the changes of many serum metabolites. Collectively, these findings suggest that specific intestinal bacteria may significantly contribute to the regulation of host metabolism, which are in good agreement with the former reports that gut microbiota is involved in the regulation of multiple host metabolic pathways playing an important role in human health and disease.^47^

## Discussion

Growing evidence indicated the importance of gut microbiota in pathophysiology of neuropsychiatric disorders. However, the causal relationship between them has still not been established. In this study, we found that gut dysbiosis in early-life impaired neurodevelopment by remodeling serum metabolome, and observed that increasing serum 4-methylphenol induced ASD-like behavior deficits. Postnatal early-life is a critical period for microbiota colonization in both mice and humans. Antibiotics constitute an important cause of early-life dysbiosis by their wide application. In addition, multiple factors including delivery mode, breastfeeding, and diet also remarkably affect the homeostasis of gut microbiota. For example, infants delivered by cesarean section reduced the diversity of gut bacteria and increased the risk of developing ASD and attention deficit when compared to those delivered vaginally.^48,49^ Consumption of a western diet impaired cognitive function and increased anxiety through modifying gut microbiota.^50^ Given the vulnerability of gut microbiota in early-life and the gut-brain bidirectional interaction, it is not surprising that many adult neuropsychiatric disorders have their roots in this critical developmental window.

Here, our results suggest that some bacteria may be especially important for human health and disease. We found that *Alistipes, Megamonas, Desulfovibrio*, and *Butyrimimonas* increased their abundance in antibiotic-treated mice, while other bacteria showed the opposite trend, such as the *Dubosiella, Parasutterella*, and *Faecalibaculum*. Coincidentally, the specific changes of such bacteria have been reported in patients with major depressive disorder, autism, memory deficit, and gastrointestinal disorders.^24–27^ It is important to note that these bacteria positively or negatively co-vary with the behavioral outcomes of our dysbiosis mice. While reconstitution of normal microbiota rescued the behavior deficits, indicating a causal role for gut microbiota in modulating CNS development. All members of the microbiota do not have the same effect on the host, some bacteria may influence the host by specialized mechanisms, such as promoting inflammation and producing bioactive metabolites. Therefore, understanding the influence of specific bacterial populations on brain development will pave the way for clinical intervention and treatment of psychiatric disorders related to gut dysbiosis.

In this study, we demonstrated that gut dysbiosis in early-life impaired hippocampal neurogenesis that was restored by reconstructing a normal microbiota. Adult neurogenesis is an important process in regulating brain function and behavior. Reduction of adult neurogenesis has been associated with several psychiatric disorders, such as anxiety, depression, and memory loss.^51^ Neurogenesis has been reported to be orchestrated by several intrinsic and extrinsic factors, such as inflammatory cytokines, neurotransmitters, physical activity, and dietary intake.^52^ In addition, Ly6Chi monocytes were reported to be involved in modulating hippocampal neurogenesis in ABX-treated mice.^53^ Indoles, microbial metabolites of dietary tryptophan, were found to increase adult neurogenesis via the aryl hydrocarbon receptor pathway.^54^ Gut microbiota from chronic stress-treated mice decreased neurogenesis in healthy mice by impairing the Trp metabolism.^55^ In this study, the effect of gut dysbiosis on neurogenesis may be driven by multiple aspects, such as immune cell infiltration and circulating metabolites changes.

Previous evidence suggested that gut microbiota and prebiotics modulate mRNA expression of genes implicated in lactate shuttle in hippocampus.^56^ In this study, transcriptomic analyses indicated gut dysbiosis in early-life leads to molecular changes in hippocampus. We found that both of the genes *Lcn2* and *Lrg1* were overexpressed in hippocampal neurons following disruption of gut microbiota, and our results further demonstrated that elevating the level of 4-methylphenol in serum, a metabolite produced by gut microbiota, may mediate the upregulation of these genes. Previous evidence showed that the expression of LCN2 could be strongly induced in brain by intraperitoneal injection of bacterial lipopolysaccharide (LPS).^57^ High levels of LCN2 in hippocampal neurons were previously shown to cause a decrease in spine density and the proportion of mushroom spines.^32^ LRG1, a brain-enriched LRR protein, has been found to inhibit BDNF-induced dendrite morphology in hippocampal neurons.^58^ Overexpression of LRG1 in the brain leads to memory impairment.^33^ Taken together, our results not only suggest that the overexpression of LCN2 and LRG1 contributes to the behavior deficits and changes in dendrite morphology after dysbiosis but also show that gut microbiota participates in regulating their expression.

IEGs, known as molecular markers of neural activity in the brain, are rapidly induced by a variety of external stimuli and behavioral experience. They are believed to be crucial for synaptic plasticity, hippocampal memory, and long-term changes in mood.^59^ Decreased hippocampal IEGs expression by infusion of antisense oligonucleotide has been reported to impair learning and memory.^60^ In this study, we found that antibiotic treatment-induced dysbiosis suppressed the increased hippocampal IEGs expression after social test (Figure 5E, FigureS4). Our result further suggest that bacterial metabolites may mediate the changes, such as the 4-methylphenol (Figure 9G, FigureS8A). The effect of gut microbiota on IEGs expression following social behavior test was also described in a recent article. It shows that expression of cFos was increased after a social encounter in hippocampal DG in GF mice and ABX or AVNM mice, compared with the wild-type control.^61^ Such difference in the expression of IEGs may come from the different mouse model: GF mouse has no living microorganisms in or on them from birth. While the ABX or AVNM (a combination of 1 g/L ampicillin, 0.5 g/L vancomycin, 1 g/L neomycin, and 0.5 g/L metronidazole) treatment was frequently reported to completely removing gut microbiome.^61,62^ In our study, the newborn mice were treated with a single antibiotic (ampicillin or vancomycin) to induce dysbiosis (Figure 2) rather than complete loss of intestinal flora. The other reason may lie in the fact that the expression of IEGs is regulated by multiple mechanisms, such as histone acetylation and deacetylation, transcription elongation factors, serum response factor, and cAMP response element-binding protein.^59,63^ The abnormal expression of IEGs may significantly affect neuronal function in critical neural circuits. Indeed, down-regulation of IEGs has been found during neurodegeneration, brain injury, pathological stimuli, and neuronal apoptosis.^64^

Consistent with the roles of gut microbiota in regulating nutrient absorption and metabolic homeostasis,^65^ we demonstrated the large effect of the microbiome on host plasma biochemistry, and found that serum metabolomic changes contributed to the impairment of hippocampus and behavior. Accumulating evidence suggest that gut microbiota produces a variety of bioactive small molecules during metabolism of food and xenobiotics, many of which show a direct role in human health and disease,^47^ even a single metabolite could be beneficial or toxic to host. For example, the microbial butyrate has been reported to enhance intestinal barrier function and mucosal immunity;^66^ while the higher circulating trimethylamine-N-oxide (TMAO) induced cognitive dysfunction by increasing neuroinflammation in hippocampus.^67^ In this study, we revealed that increasing the level of 4-methylphenol in blood, a bacteria metabolite elevated in the urinary of children with ASD, is sufficient to induced ASD-like behavior impairment by decreasing social activity and increasing anxiety in naive mice. Interestingly, a recent study shows that 4-methylphenol treatment-induced changes in microbiota composition and ASD core behavioral symptoms in mice.^68^ Taken together, these findings may pave the way for the treatment of ASD by intervening the gut microbiota and 4-methylphenol production.

In this study, our results show that early-life gut dysbiosis induced by two different antibiotics leads to similar hippocampal dysfunction and behavioral damage in mice, and suggest that changes of serum metabolites may mediate the effect. However, there may be differences in its potential mechanism. Indeed, we should note that there are differences in the composition and structure of gut microbiota and hippocampal transcriptional changes between the two groups of antibiotic intervention mice. More importantly, the serum metabolomics of Amp-treated and Van-treated mice were significantly different, although they have some common differential metabolites. In Van-treated mice, the level of 4-methylphenol in serum increased significantly. Our results further suggest that increasing serum 4-methylphenol impaired hippocampal plasticity and induced ASD-like behavior deficits. However, 4-methylphenol decreased to some extent in the serum of Amp-treated mice, which implies that other metabolites or metabolite combinations contribute to the behavior impairment. In Amp-treated mice, lipids, and lipid-like substances account for a large part of serum differential metabolites (FigureS7B), especially the molecules belonging to glycerophospholipids and eicosanoids (Figure 8B). Glycerophospholipids, which are highly enriched in the brain, were found to be key components of the neural membrane and involved in dendrite branching and outgrowth.^69^ Evidence has shown that polyunsaturated fatty acids used by brain for synthesizing glycerophospholipid are transported there from the gastrointestinal tract.^70^ Thus, serum glycerophospholipids may contribute to establishing correct dendritic morphologies. Alterations in glycerophospholipid composition have been reported in acute brain injuries, Alzheimer's disease, and schizophrenia.^70^ Eicosanoids are oxygenated derivatives of 20-carbon polyunsaturated fatty acids known to play important roles in many physiological events, which are mainly involved in regulating inflammation and immunity.^71^ In addition, other metabolites may be also responsible for the abnormal neurological function observed in Amp-treated mice (Figure 8B). For example, the glutamic acid and aspartic acid as neurotransmitters were reported to play an important role in brain functions, and excessive intake of them could cause damage to the nervous system;^72^ the metabolite styrene was reported to have an obvious effect on dopamine and serotonin in brain;^73^ L-pipecolinic acid, a lysine metabolite, was shown to be involved in synaptic transmission and induce apoptosis in neuronal cells.^74^ Collectively, the phenotypic damage and hippocampal dysfunction in antibiotic-treated mice may be caused by the changes of a variety of serum metabolites.

Our results suggest that gut dysbiosis in early-life impaired hippocampal plasticity and behaviors by remodeling serum metabolomics. Many studies show that hippocampus is particularly vulnerable to changes in microbial composition. Indeed, molecular changes in the prefrontal cortex and amygdala were also observed in GF mice. Are there any changes in the prefrontal cortex, amygdala, and other brain tissues of antibiotic-treated mice? The prefrontal cortex (PFC) plays an important role in modulating cognition, anxiety, and social behaviors. Previous studies showed that GF mice alters adult PFC myelination,^13^ microRNA expression,^75^ and lipid metabolism.^76^ In fact, we made some explorations about whether the expression of myelin genes and BDNF were altered in our antibiotic-treated mice; however, no significant difference in these genes was found compared with wild-type control (FigureS9). Unfortunately, we lack in-depth research on whether early-life gut dysbiosis leads to structural and functional changes in the PFC. The amygdala, a core brain area involved in emotion processing and regulation, is another potential target of intestinal flora imbalance. Emerging evidences suggested that GF status in mice resulted in changing the dendritic morphology,^77^ altering the expression of immediate-early genes and neural activity associated genes^4^ in amygdala. We are very interested in this issue about the potential changes in PFC and amygdala, and more systematic research on antibiotic-treated mice may be conducted in the future.

Taking all data together, we show that postnatal early-life represents critical periods during which gut dysbiosis impaired neurodevelopment by remodeling serum metabolomics. Our findings emphasize the important role of microbial metabolites in early-life brain development and behavior, and highlight the potential microbiome-mediated therapies for treatment of neurodevelopmental disorders. However, further studies are needed to reveal the exact mechanisms underlying gut-brain communication and investigate the role of specific bacteria in brain development.

## Methods and Materials

### Animals

The C57BL/6 J mice were bred and reared under the same conditions following the institutional guidelines and the Animal Care and Use Committee of the animal core facility at Huazhong University of Science and Technology, Wuhan, China. Mice were housed in groups of three to five mice/cage with a 12-h light/dark cycle at a consistent ambient temperature (21 ± 1°C) and humidity (50 ± 5%).

## Experimental group and time lines

The new-born mice (P1) were treated with antibiotics (100 mg/kg ampicillin or 50 mg/kg vancomycin) or vehicle (distilled water) by oral gavage daily between 9:00 and 10:00 AM (no more than 30 μL) for four weeks (P1-P28) using the feeding needle (24 Gauge, 1-inch needle length, 1.25 mm ball diameter) according to the methods previously described.^78^ In order to avoid potential degradation of antibiotics, the antibiotic solution was renewed every day. Following the last antibiotic gavage, the offspring were separated from the dams and housed 3–5 per cage and received regular drinking water. The feces of the mice were collected at the day P42 for 16S rRNA sequencing to investigate whether antibiotic exposure led to durable changes in gut microbiota. Then, behavioral testing was conducted in the order of OFT (P43), EPM (P45), MWM (P47-P54), and three-chamber sociability test (P56) with one day interval between testing (Figure 1). Animals were put back into their home cages following the social behavior test for 1.5 h to induce the expression of IEGs, and then the mice were perfused or the brains were collected immediately for further analysis.

For fecal microbiota transplantation (FMT), a fecal microbiota transfer protocol was conducted after the final antibiotic gavage.^79,80^ In order to prepare the microbiota suspension, 0.3 g fresh feces pellets were collected daily between 9:00 and 10:00 AM from four-week-old C57BL/6 J SPF mice, and they were resuspended in 3 ml reduced PBS (sterile PBS with 0.2 g /L Na_2_S and 0.5 g/L cysteine) by vortex. The supernatant was collected by centrifuging at 500 g for 5 min, and then each recipient mouse was daily administered with 200 μL of supernatant within 10 min by oral gavage for two weeks (P28-P42). At the same time, the control mice (wild-type C57BL/6 J mice) that did not receive FMT were daily given with 200 μL of reduced PBS by oral gavages for two weeks (P28-P42) in order to match the stress of gavage operation. After colonization, the FMT-treated mice were reared in a gnotobiotic environment with normal drinking water. Then, animals were subjected to a series of behavioral tests mentioned above (P43-P56). After the social behavior test, the mice were returned to their home cages for 1.5 h to induce the expression of IEGs, and then mice were perfused or the brain tissues were collected immediately.

For 4-methylphenol (*p*-cresol)-treated group: wild-type mice (4-week-old C57BL/6 J SPF mice) were injected i.p. with saline or 4-methylphenol (35 mg/kg) daily for two weeks according to the methods described by Gigi Tevzadze et al. (2020) with some modifications.^81^ Then, animals were subjected to a series of behavioral tests in the order of OFT, EPM, MWM, and three-chamber sociability test (P43-P56) with one-day interval between testing. The animals were sacrificed at 1.5 hours after the social behavior experiment as mentioned above.

## 16S rDNA Gene Sequencing Analysis

Fresh feces were collected at the day P42 in sterile tubes and immediately stored at −80°C. Total genomic DNA was extracted using the CTAB/SDS method. The V3-V4 region of the 16S rRNA gene was amplified using barcoded primers for the Illumina platform, as previously described.^82^ Samples were pooled and sequenced with Illumina NovaSeq platform (NOVOGENE Company Limited, China) and 250 bp paired-end reads were generated. Using QIIME and UCHIME, sequences were quality filtered and trimmed. The operational taxonomic units (OTUs) were chosen based on 97% sequence similarity to Silva Database. Phylogenetic relationship construction was conducted using the MUSCLE software, Alpha diversity and Beta diversity were calculated by QIIME software (V1.9.1).

## Behavioral testing

A battery of behavioral tests was started in the order of OFT, EPM, MWM, and three-chamber sociability test with one-day interval between testing when the mice were 6 weeks of age. The mice were allowed to habituate to the testing room for at least an hour before test commencement. Test chambers were cleaned with 70% ethanol and aired for 3 min after each animal. Experimenters were blind to the treatment group. All behavioral tests were recorded by a video camera, and data analysis was performed by using automated Ethovision video tracking software (EthoVision XT, Noldus).

OFT: spontaneous motor activity was assessed over 10 min in an arena (45 cm × 45 cm × 60 cm). The total distance traveled was recorded to evaluate the locomotor activity, and the time spent traveled in the center and the peripheral zone was used to analyze the anxiety-like behavior.

EPM test: The EPM apparatus was comprised of two open arms (30 cm × 6 cm) and two closed arms (30 cm × 6 cm × 15 cm) elevated at 50 cm off the ground, Mice were individually placed in the intersection of the four arms, facing an open arm, and allowed to explore for 5 min. The following parameters were recorded to assess anxiety-like behavior: time spent, distance traveled, and the number of entries in the open arms.

MWM test: The Morris water maze is a test for assessing spatial learning and memory for rodents. This test was conducted as previously published with a few modifications (Morris water maze: procedures for assessing spatial and related forms of learning and memory). Each mouse was given four trials a day for 6 consecutive days. The time is taken to locate the escape platform (escape latency) was measured. After the training trials, the mice could rest for one day and then were given a probe trial. During the probe trial, the platform was removed and each animal was allowed 90 s to search the pool. The amount of time and distance that each animal spent in the target quadrant was recorded and analyzed.

Three-chamber sociability test: sociability (spending more time in the chamber containing a stranger mouse than in the empty chamber) and social novelty (spending more time in the chamber containing novel mouse than in the chamber containing the familiar mouse) were evaluated using a three-chamber social test system. Briefly, in the sociability test, experimental mice were placed into the central chamber and allowed to explore for 5 min. Following the habituation phase, a stranger mouse (Stranger I) was introduced into the wire cage in the left or right chamber, while an identical empty wire was placed in the opposite chamber. The subject mouse was given 5 min to explore the entire social test arena. In the third phase of the test, a new stranger (Stranger II, novel mouse) was introduced into the previously empty wire cage, and the already explored mouse (Stranger I, familiar mouse) remains inside its cage. The subject mouse explores the entire social test arena for a 5-min session. Each session was recorded by a video‐tracking system, and the amounts of time spent in each chamber were analyzed. Based on the amount of time spent in each session, the ‘sociability index’ and the ‘social novelty index’ were calculated according to the following formulas:^83^ Sociability index = ((Time_stranger_/ Time_stranger_ + Time_empty_) ×100)-50. Social novelty index = ((Time_novel_/ Time_novel_ + Time_familar_) ×100)-50.

## Golgi staining

Golgi staining was performed using the FD Rapid GolgiStain™ Kit (Fdneurotech, Columbia, USA) according to the manufacturer’s instructions. Briefly, the freshly dissected brains (n = 3 for each group) were rinsed in double distilled water and then immersed in the solution A/B, and stored at room temperature for two weeks in the dark. Then, the brains were transferred to Solution C and stored at 4°C for at least 72 hours. The brains were rapidly frozen and cut into 150 μm sections using a vibrating microtome (Leica, Germany). After staining, the sections were imaged using an upright bright-field microscope.

## EdU treatment

EdU (5-ethynyl-2′-deoxyuridine) is a thymidine analog optimal for labeling proliferating cells in the nervous system. Mice were intraperitoneally injected with 25 mg/kg of EdU for 3 consecutive days, and they were examined one weeks later. The slices of brains were prepared following the standard immunohistochemical protocols, EdU labeling was detected by using the Click-iT™ EdU Imaging Kit with Alexa Fluor™ 488 dye (Invitrogen) according to manufacturer’s instructions.

## Hippocampal LTP induction

The brain of 2-month-old mice was dissected, and then transverse hippocampal slices (400 μm) were prepared by using a tissue chopper. Slices were immersed in ice-cold artificial cerebrospinal fluid (ACSF: 124 mM NaCl, 25 mM NaHCO_3_, 10 mM glucose, 3.35 mM KCl, 2 mM MgSO_4_, 2 mM CaCl_2_ and 1.25 mM NaH_2_PO_4_), bubbled with 5% CO_2_, 95% O_2_. For recording, slices were placed into a chamber maintained at 32°C and continuously perfused (2.5 ml/min) with oxygenated artificial cerebrospinal fluid^84^. To induction of LTP, the DG area was stimulated by single monopolar pulses at 30 s intervals and the field excitatory postsynaptic potentials (fEPSPs) were recorded in the CA3 area with ACSF-filled micropipettes (resistance value:2–4 MΩ). The stimulation intensity that evoked 30% of the maximal slope was used for the test pulses and LTP induction protocol. Following a minimum of 20 min of stable baseline recording, LTP was induced using three consecutive trains (500 ms) of stimuli at 100 Hz separated by 10 s.

## Immunohistochemistry

Mice were deeply anesthetized and perfused with 4% paraformaldehyde (PFA) in PBS. Brains were dissected out, post-fixed in 4% PFA overnight at 4°C, and then cryopreserved with 30% sucrose. Sections (30 μm) were cut using a cryostat (Leica), and then permeabilized and blocked with 3% BSA in 0.1% Triton X-100 containing PBS. Subsequently, the slices were incubated with primary antibodies at 4°C for 24 h. After extensive washing, the fluorescently conjugated secondary antibodies in the blocking solution were applied, followed by nucleus staining with DAPI. The slices were visualized on Zeiss LSM800 confocal microscopes.

Quantification of hippocampal neuron number was performed on the left hemisphere using systematic random sampling of every 10th section throughout the entire hippocampus. Coronal hippocampal sections stained for NeuN were selected for further analyses. The hippocampal subfields were delineated based on the Franklin and Paxinos mouse brain atlas. The Cavalieri’s principle was applied to calculate the volumes of CA1 and the DG, and then the total numbers of neurons were calculated as previously described.^85^ Cells with typical neuronal morphology were counted using a 100x oil objective. Data are shown as the mean number of cells in the left hippocampus in five animals per group.

## RNA Sequencing and analysis

RNA from the hippocampus was extracted using TRIzol (Invitrogen). RNA integrity was assessed using the Bioanalyzer 2100 system (Agilent Technologies), and high-quality samples were selected for library preparation. After cluster generation, the library preparations were sequenced on an Illumina Novaseq platform ((NOVOGENE Company Limited, China) to obtain 150 bp paired-end reads. The clean paired-end reads were aligned to the reference mouse genome using Hisat2. Differential expression analysis was performed using the DESeq2 R package (1.30.1) with standard settings. Genes with an FDR adjusted *P*-value (Benjamini and Hochberg’s approach) <0.1 were assigned as differentially expressed. Corrected *P*-value of 0.1 and absolute foldchange of 2 was set as the threshold for significantly differential expression. Differentially expressed genes (corrected *P*-value of 0.1) were selected for GO and KEGG enrichment analysis by the cluster profile R package, GO terms and KEGG pathways with *P*-value less than 0.01 were considered significantly enriched.

## Western blotting

Brain tissues were quickly dissected following decapitation and stored at −80°C. Samples were homogenized in protein lysis buffer containing protease inhibitor cocktail (Roche). The protein was estimated by the Bradford method, denatured, and separated by SDS-polyacrylamide gel electrophoresis, and then transferred onto nitrocellulose filter (NC) membranes (Whatman). Membranes were blocked in 3% bovine serum albumin in TBS-0.1%Tween-20 for 1 h at room temperature and incubated in primary antibodies solution overnight at 4°C. After extensive washing, membranes were incubated with IRDye®800CW secondary antibodies (LI-COR Biosciences) for 2 h at room temperature and then scanned using the Odyssey® CLX Infrared Imaging System. The protein bands were quantified by using the ImageJ software. The antibody used in this study is illustrated in Supplementary Table 1.

## Real-time RT-PCR

Tissues of the hippocampus and prefrontal cortex were stored in RNAlater (Qiagen) at −80°C until tissue processing. Total RNA was isolated using TRIzol (Invitrogen) according to the manufacturer’s instruction. Quantification RT-PCR was carried out using the SYBR Premix Ex Taq II Kit in an ABI PRISM 7000 Sequence Detection System. The mRNA expressions were calculated via relative quantification and analyzed by 2**^−^**^ΔΔCT^ formula and normalized to the housekeeping gene (GAPDH or ACTB). Primer sequences were illustrated in Supplementary Table 2.

## Metabolomics data analysis

Blood samples were centrifuged at 1,500 g for 10 min 4°C to collect serum. Serum was then sent to NOVOGENE Company Limited (Beijing, China) for metabolite extraction and liquid chromatography-tandem mass spectrometry analysis. Then, metabolites were annotated using the KEGG database, HMDB database, and LIPID Maps Database. T-test was applied to calculate the statistical significance (*P*-value). The metabolites with VIP > 1 and *P*-value< 0.05 and fold change>2 were considered to be differential metabolites.

## 4-methylphenol treatment

4-methylphenol (Sigma-Aldrich) was dissolved in dimethyl sulfoxide (DMSO) (1000-fold concentrated) and then diluted in culture medium to give a final concentration of 60 μM. Hippocampal neuronal cultures were prepared from 16-day-old embryonic C57BL/6 J mice, and then cells were cultured. Four days after plating, cells were treated with 4-methylphenol at 60 μM for 48 hours as well as DMSO as a control. After that, treated cells were harvested for subsequent assays. For in-vivo experiment, 4-week-old C57BL/6 J SPF mice were injected i.p. with saline or 4-methylphenol (35 mg/kg) daily for two weeks according to the methods previously described.^81^

## Statistical analysis

Data were collected from at least three biological replicates or three independent experiments and were presented as mean ± SEM. According to the distribution of data, significance was calculated using unpaired two-tailed *t*-test or one-way ANOVA with Bonferroni post hoc analysis, where appropriate. Significant differences were indicated in the figures by *P < 0.05, **P < 0.01, ***P < 0.001. In RNA-Seq analysis, and FDR adjusted *P*-value of <0.1 (*P*adj, Benjamini-Hochberg method) was set for selections of significantly differentially expressed genes as previously reported. Differences were considered statistically significant at *P* < 0.05 unless otherwise indicated.

## Supplementary Material

Supplemental Material

## Data Availability

The raw data that support the findings of this study have been deposited into CNGB Sequence Archive (CNSA) of China National GeneBank DataBase (CNGBdb) with accession number CNP0002032 (16S rRNA Gene Sequencing), CNP0002038 (RNA Sequencing), CNP0002054 (Metabolomics).
