# Supplementary material for: Gut dysbiosis impairs hippocampal plasticity and behaviors by remodeling serum metabolome
Source: Gut Microbes. 2022 Jul 24;14(1):2104089. doi: 10.1080/19490976.2022.2104089 (PMC9327780; doi:10.1080/19490976.2022.2104089)
Supplement: Supplemental Material [file KGMI_A_2104089_SM8576.pdf]

Supplemental Information

Supplementary Figures

Fig.S1

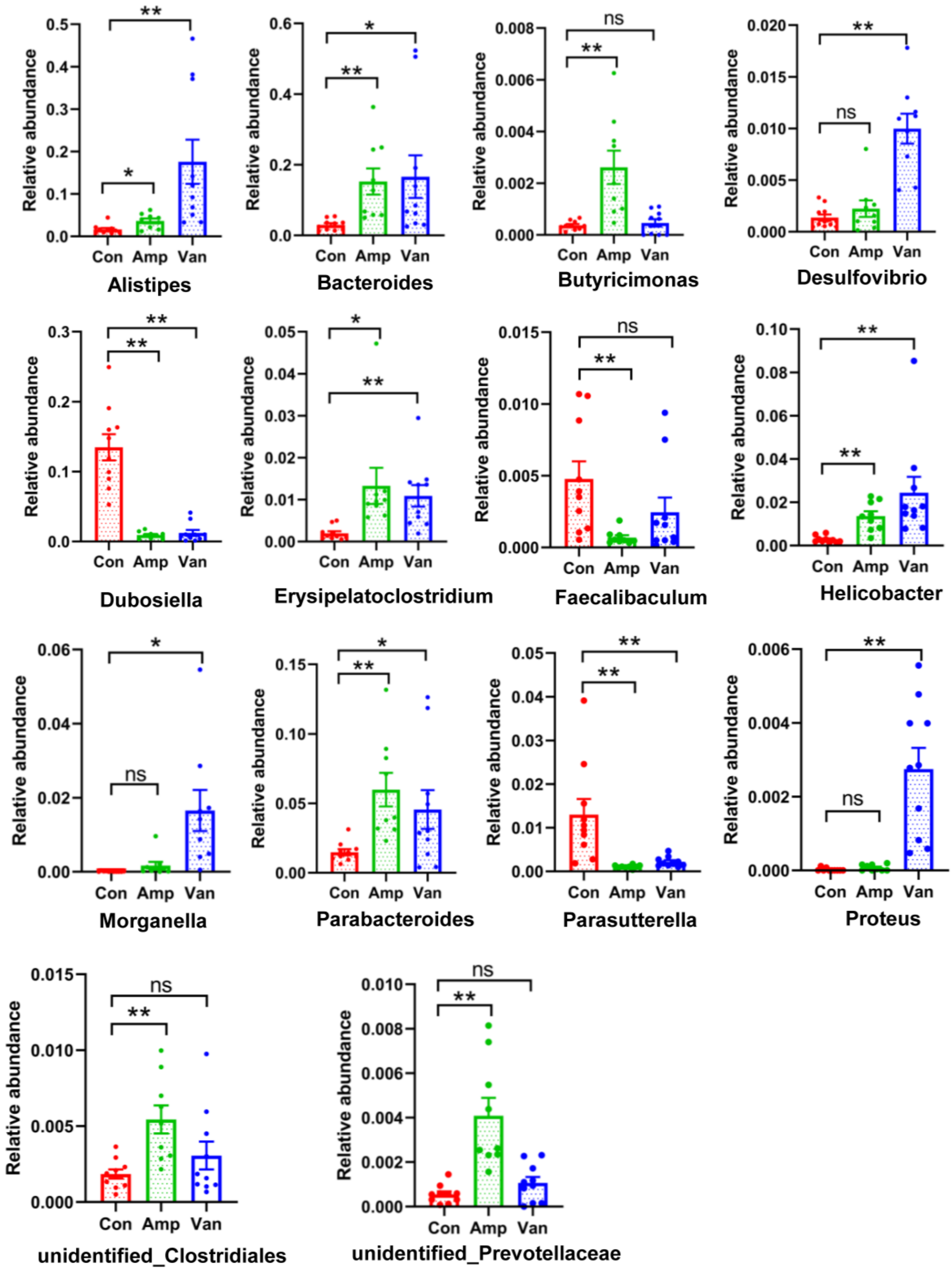

Figure S1: Relative abundance of representative bacteria genera that were significantly altered following disruption of gut microbiota.

The *Alistipes*, *Megamonas*, *Desulfovibrio*, and *Butyrimimonas* increased their abundance in antibiotic-treated mice, while other bacteria showed the opposite trend, such as *Dubosiella*, *Parasutterella* and *Faecalibaculum*. The specific changes of such bacteria have been reported in patients with major depressive disorder, autism, memory deficit, and gastrointestinal disorders. Data are presented as mean ± SEM, n=9-10/group, unpaired two-tailed *t*-test, **P*<0.05, ***P*<0.01, ns: no significance.

Fig.S2

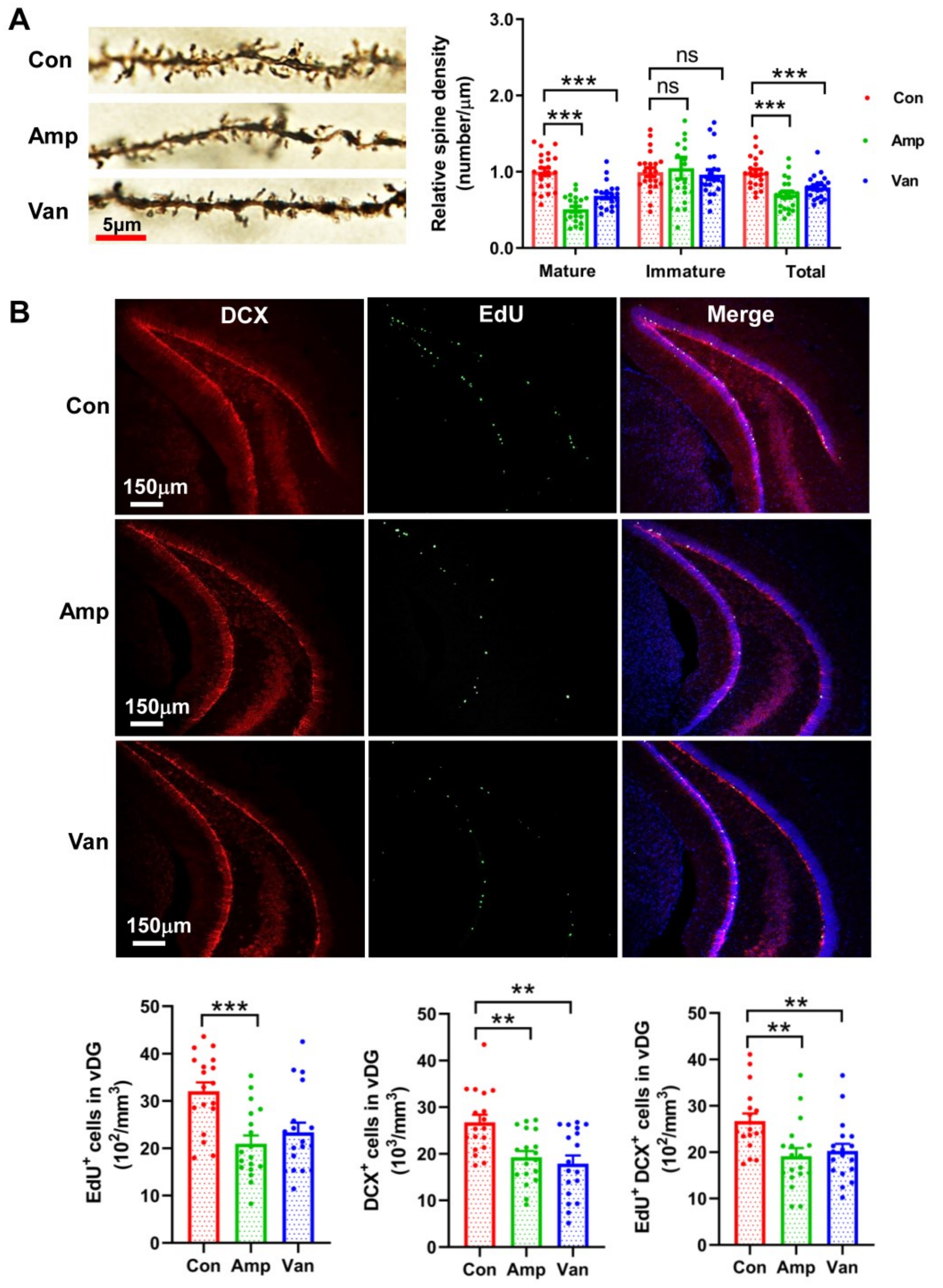

Figure S2: Disruption of gut microbiota impairs hippocampal CA1 spine maturation and adult neurogenesis

(A) Golgi staining was used to visualize apical dendritic processes in the CA1 of the ventral hippocampus, the mature-appearing spine (mushroom spine) decreased following disruption of gut microbiota. N=3/group. Data are presented as mean ± SEM, unpaired two-tailed *t*-test, ***P<0.001, ns: no significance. (B) The

representative micrographs and quantitative analysis of the cells positive for EdU and DCX in the ventral DG (vDG). EdU was used to label the proliferation of hippocampal progenitor cells, DCX was used to reflect neuronal differentiation. N=3/group. Data are presented as mean ± SEM, unpaired two-tailed *t*-test, $^{**}P<0.01$, $^{***}P<0.001$.

Fig.S3

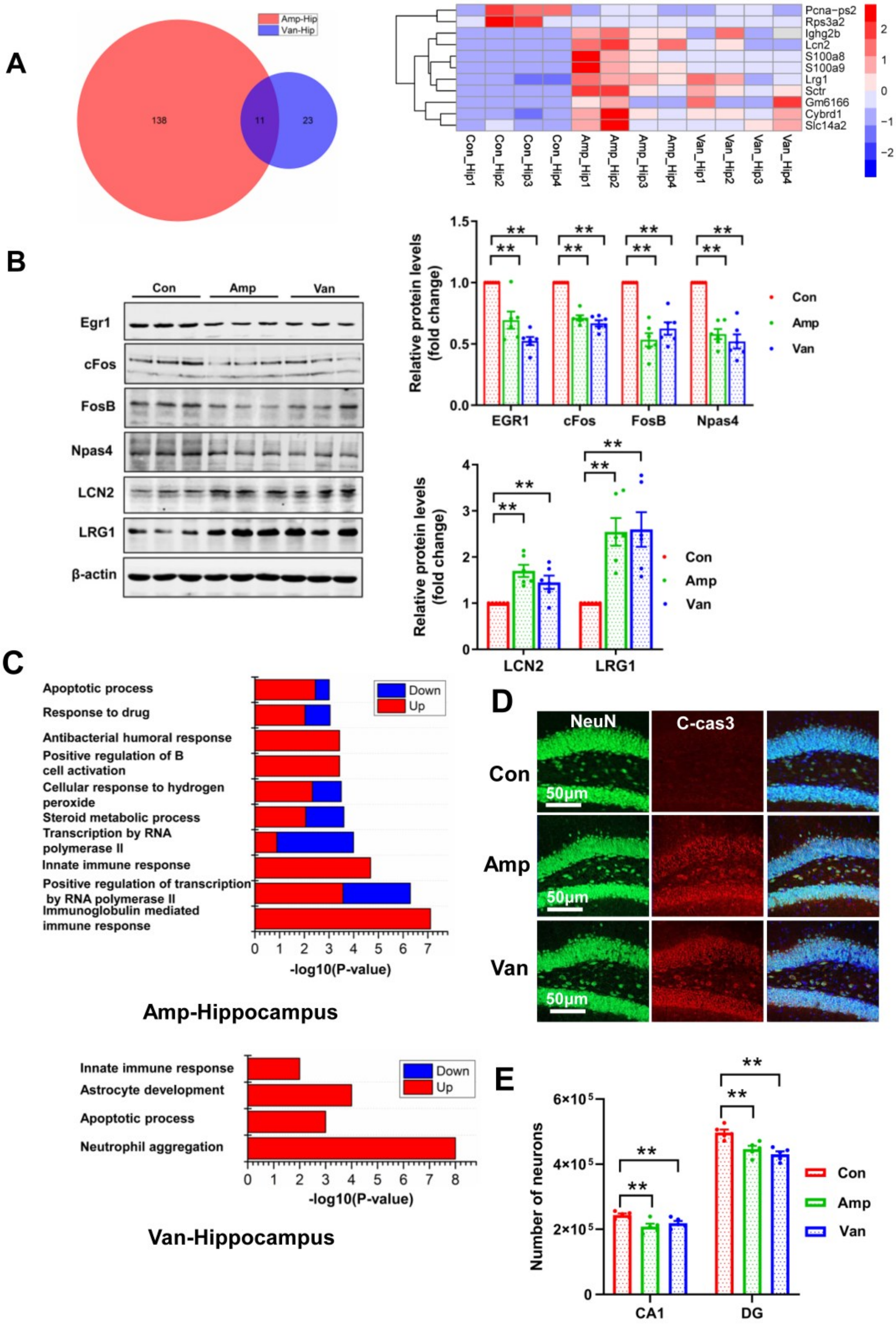

Figure S3: Gut dysbiosis alters the gene expression in the hippocampus.

(A) Heatmap of the common differential gene in hippocampus shared by antibiotic-treated mouse, FDR adjusted *P*-value of 0.1 and absolute foldchange of 2 was set as the threshold for significantly differential expression. (B) Quantitative

analysis of the expression of LCN2, LRG1 and immediate early genes, **P<0.01. (C) top biological pathways enriched for all the differential genes in antibiotic-treated mice. (D) The representative micrographs show that the cleaved caspase-3-positive cells significantly increased in the hippocampal DG following disruption of gut microbiota. N=5/group. The nuclei were stained with DAPI. (E) The representative micrographs show that early-life dysbiosis induced neuron loss in hippocampal DG and CA1. N=5/group. Data are presented as mean ± SEM, unpaired two-tailed t-test, **P<0.01.

Fig.S4

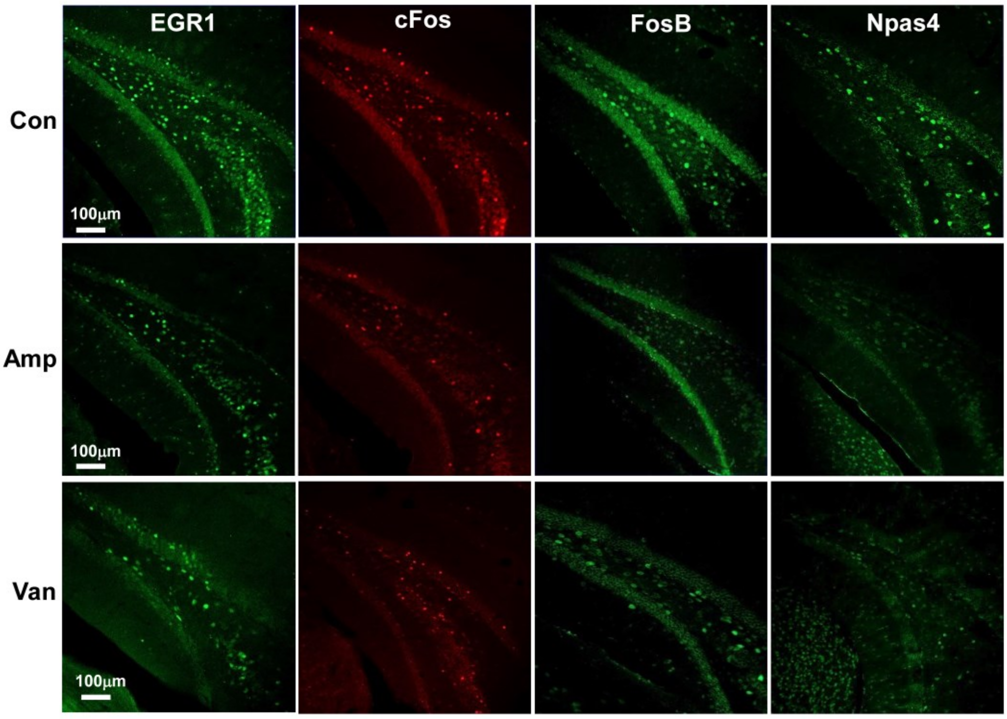

Figure S4: Antibiotic treatment induced dysbiosis suppressed the increased hippocampal IEGs expression after social test.

The representative micrographs show that antibiotic treatment resulted in an impairment the up-regulation of immediate early genes (*Egr1*, *cFos*, *FosB*, and *Npas4*) induced by social behavior test. N=5/group.

Fig.S5

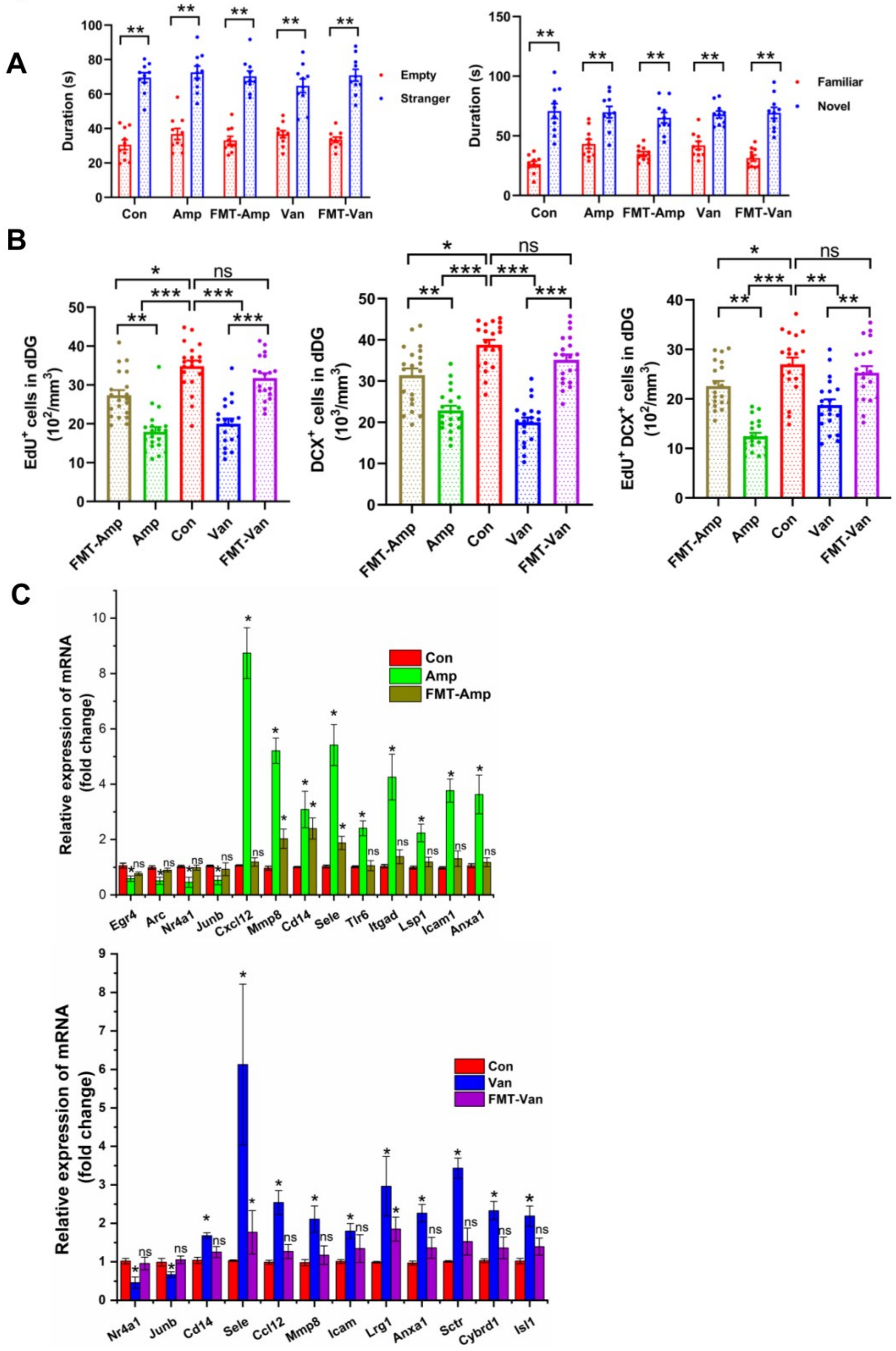

Figure S5: Reconstitution with normal gut flora rescues the behavior deficit, adult neurogenesis, and the expression of differential genes in antibiotic-treated mice.

(A)The interaction zone duration in the three-chamber sociability test. N=9-10/group.

Data are presented as mean ± SEM, unpaired two-tailed *t*-test, $^{**}P<0.01$. (B) Quantitative analysis of the cells positive for EdU, DCX and double-labeled DCX-EdU cells in the DG. N=5/group. Data are presented as mean ± SEM, unpaired two-tailed *t*-test, $^{*}P<0.05$, $^{**}P<0.01$, $^{***}P<0.001$, ns: no significance. (C) Real-time RT-PCR analysis shows that reconstituting healthy microbiota rescued the expression of differential genes in antibiotic-treated mice. The mRNA expressions were calculated via relative quantification and analyzed by $2^{-\Delta\Delta CT}$ formula and normalized to the housekeeping gene GAPDH. Data are presented as mean ± SEM, unpaired two-tailed *t*-test, $^{*}P<0.05$, ns: no significance.

Fig.S6

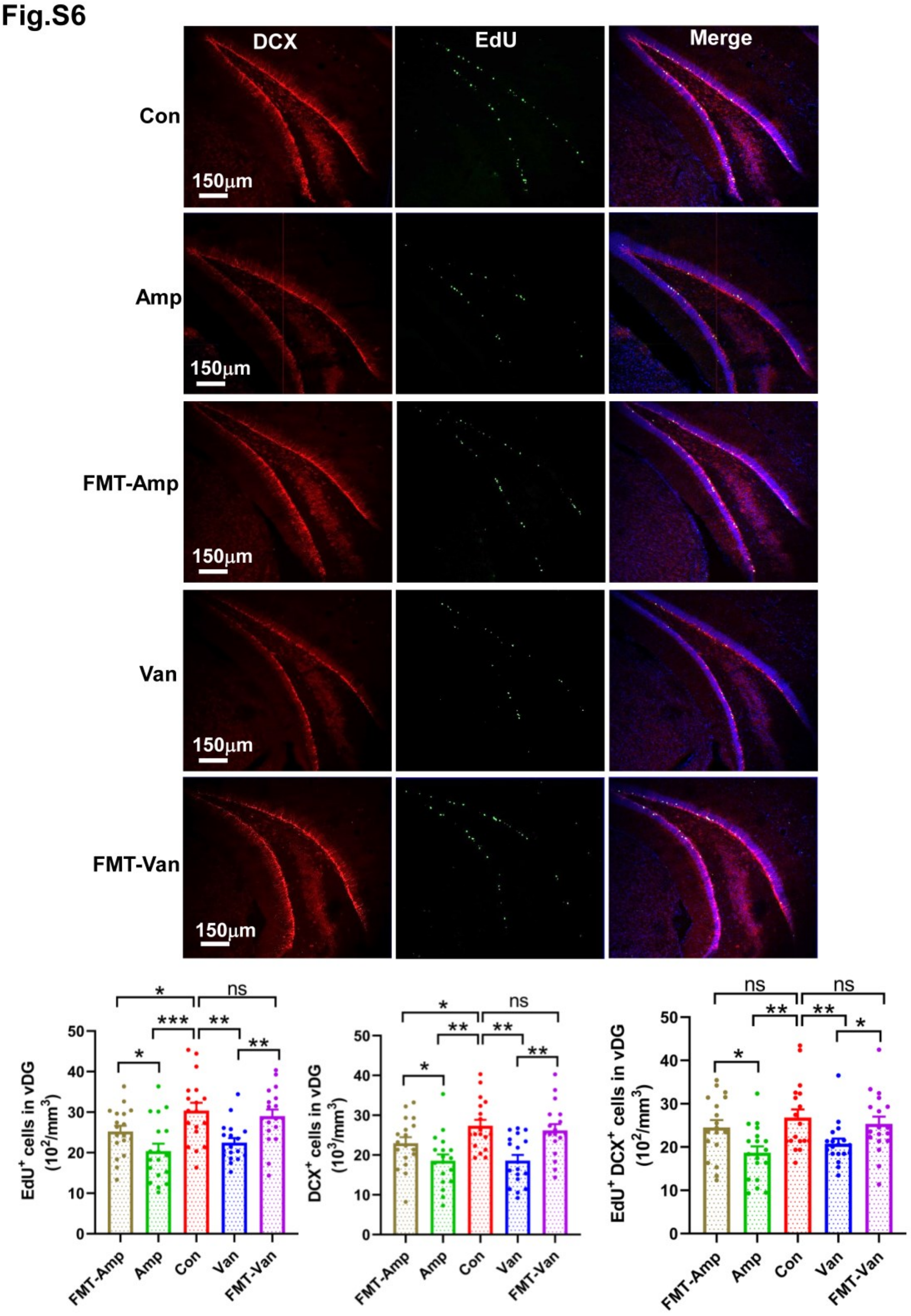

Figure S6: FMT restore the adult neurogenesis in antibiotic-treated mice.

The representative micrographs and quantitative analysis of the cells positive for EdU and DCX in the ventral hippocampus. EdU was used to label the proliferation of hippocampal progenitor cells, DCX was used to reflect neuronal differentiation. N=3/group. Data are presented as mean ± SEM, unpaired two-tailed *t*-test, **P<0.01,

***P<0.001.

Fig.S7

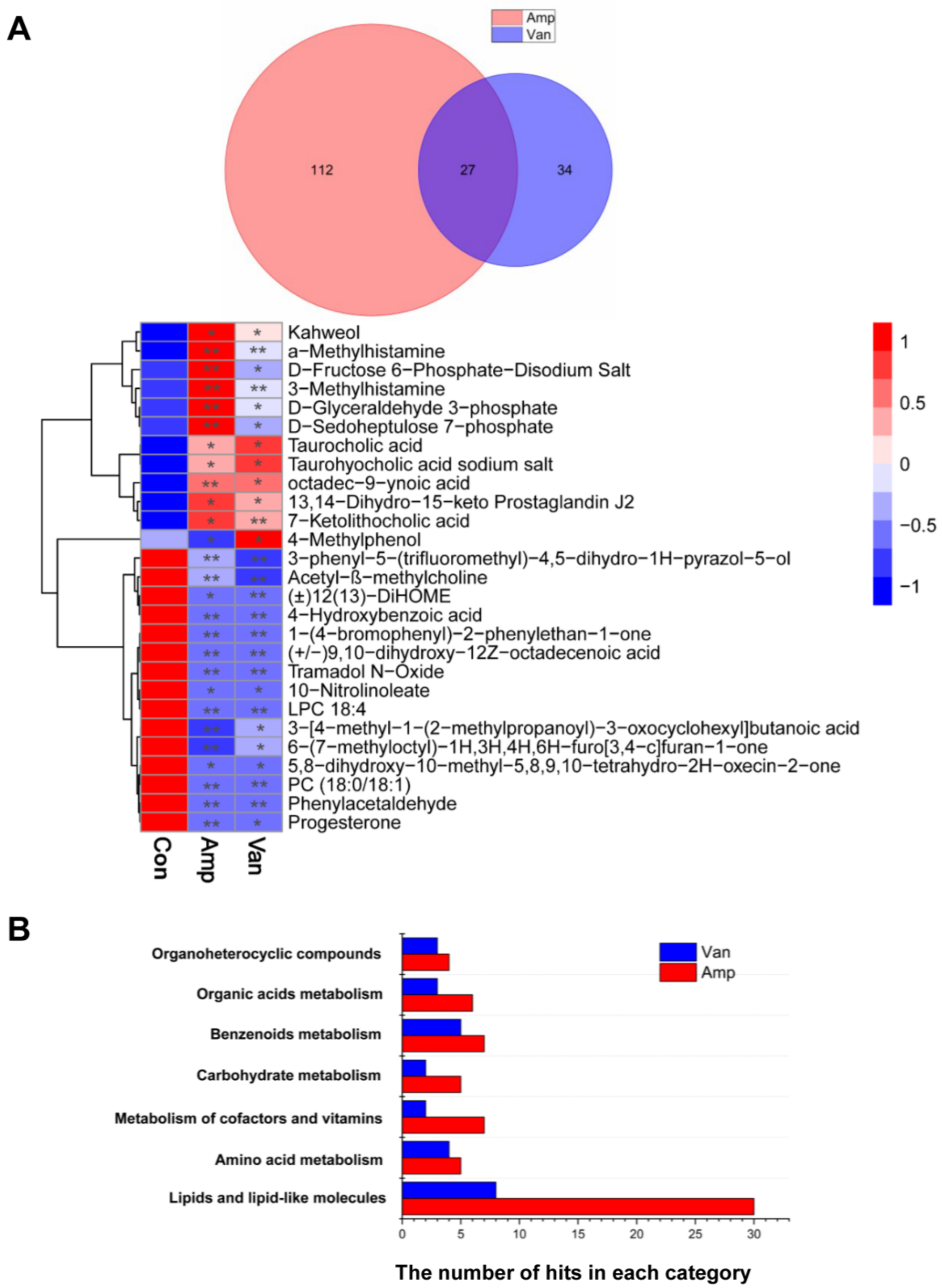

Figure S7: **The common differential metabolites in antibiotic-treated mice and**

functional annotation of metabolites.

(A) Venn diagram and heatmap show the common differential metabolites shared by Amp-treated and Van-treated mice. N=9-10/group. The metabolites with VIP > 1 and *P*-value< 0.05 and fold change>2 were considered to be differential metabolites. Unpaired two-tailed *t*-test, *P<0.05, **P<0.01. (B) Functional annotation of differential metabolites in serum. Metabolites were annotated using the KEGG database, HMDB database, and LIPID Maps Database.

Fig.S8

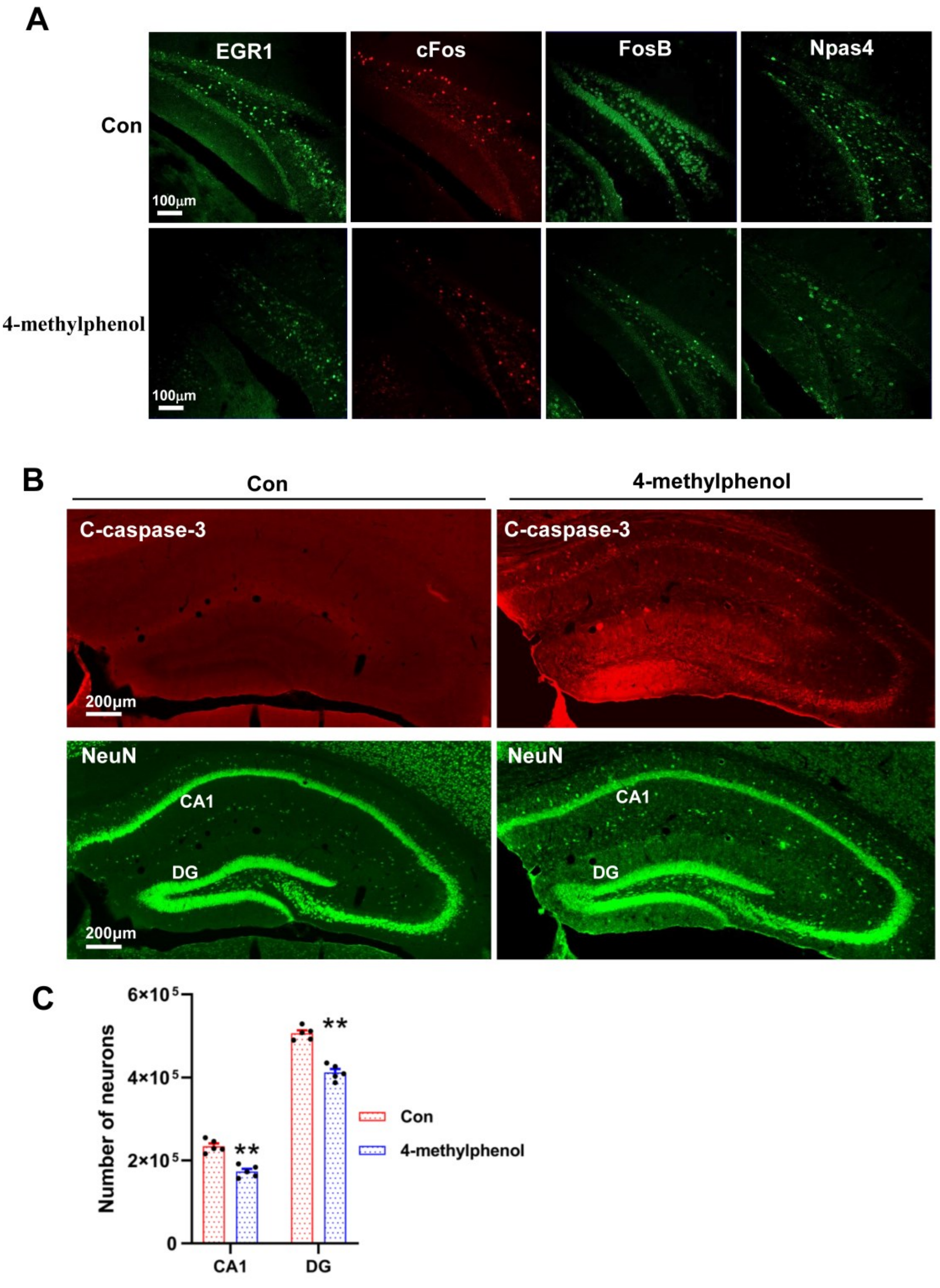

Figure S8: **The 4-methylphenol treatment suppressed the up-regulation of IEGs and induced neuron loss in hippocampus.**

(A) The representative micrographs show that 4-methylphenol treatment suppressed

the increased IEGs expression in the ventral hippocampus after social behavior test. N=3/group. (B)The representative micrographs show the hippocampal cleaved caspase-3-positive cells and NeuN-positive cells in the mice following 4-methylphenol treatment. N=5/group. (C) The 4-methylphenol treatment induced neuron loss in hippocampal DG and CA1. N=5/group. Data are presented as mean ± SEM, unpaired two-tailed *t*-test, ***P*<0.01.

Fig.S9

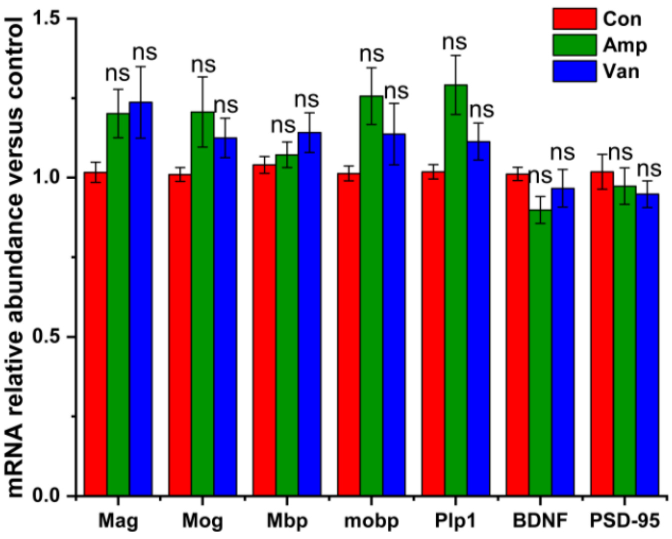

Figure S9: **Early-life gut dysbiosis did not lead to changes in the expression of myelin genes, BDNF and PSD95 in the prefrontal cortex**.

Real-time RT-PCR analysis suggests that the mRNA levels of myelin genes, BDNF and PSD95 were not changed following disruption of the gut microbiota. The mRNA expressions were calculated via relative quantification and analyzed by $2^{-\Delta\Delta CT}$ formula and normalized to the housekeeping gene GAPDH. Data are presented as mean ± SEM, unpaired two-tailed *t*-test, **P*<0.05, ns: no significance.

Supplementary Tables

Table S1: The antibody used in this study

Antibody	Supplier	Dilution ratio
Doublecortin Rabbit Polyclonal Antibody	Invitrogen-481200	IF 1:200
cFos Rat Monoclonal antibody	Synaptic Systems-226017	IF 1:400
cFos Mouse Monoclonal antibody	Santa Cruz-SC-166940	WB 1:500
EGR1 Rabbit Polyclonal Antibody	Proteintech-22008-1-AP	IF 1:200; WB 1:800
FosB Rabbit Polyclonal Antibody	Affinity-AF5010	IF 1:200; WB 1:500
NPAS4 Rabbit Polyclonal Antibody	Bioss-bs-19325R	IF 1:150; WB 1:800
NeuN Mouse Monoclonal Antibody	Millipore-MAB377	IF 1:200
LRG1 Rabbit Polyclonal Antibody	Proteintech-13224-1-AP	IF 1:150; WB 1:800
LCN2 Rabbit Polyclonal Antibody	Proteintech-26991-1-AP	IF 1:100; WB 1:500
Cleaved caspase3 Rabbit Polyclonal Antibody	CST-9661s	IF 1:200; WB 1:800
Caspase3 Rabbit Monoclonal Antibody	Proteintech-19677-1-AP	WB 1:800
CD3E Rabbit Polyclonal Antibody	Abclonal-A1753	IF 1:200
CD11b Rabbit Polyclonal Antibody	Proteintech-21851-1-AP	IF 1:200
CD45 Rabbit Polyclonal antibody	Proteintech-20103-1-AP	IF 1:200
S100A8 Polyclonal antibody	Proteintech-15792-1-AP	IF 1:200, WB 1:800
S100A9 Polyclonal antibody	Proteintech-26992-1-AP	IF 1:200, WB 1:800
GRIN1 (NMDAR1) Polyclonal antibody	Proteintech-27676-1-AP	WB 1:800
β-actin Mouse Monoclonal Antibody	Proteintech-66009-1-lg	WB 1:20000

Table S2: The primer used by real-time RT-PCR in this study

Gene	Primer sequence
Actb-F	CCACTGTCGAGTCGCGTCC
Actb-R	ATTCCCACCATCACACCCTGG
Anxa1-F	CAAAGGTGGTCCTGGGTCAG
Anxa1-R	ACTTCATCCAAGGGCTTTCCA
Arc-F	AAGCAGAGATGCTGAGGGAAC
Arc-R	TCACTGGTATGAATCACTGGG
Ccl12-F	GACACTGGTTCCTGACTCCT
Ccl12-R	ATCCAGTATGGTCCTGAAGATCAC
Cd14-F	TTTCAGAATCTACCGACCATGGAGC
Cd14-R	GTACAATTCCACATCTGCCGC
Cybrd1-F	CTCTCTCCGGGCAATCGTC
Cybrd1-R	GAGGGGTGTTTCAGGACAAA
Egr1-F	TCGGCTCCTTTCCTCACTCA
Egr1-R	GATGTCAGAAAAGGACTCTGTGG
Egr4-F	TTCTCTCCAAGCCCACCGAA
Egr4-R	AGCTCAAGAAGTCGCCTCCA
cFos-F	AGAGCGGGAATGGTGAAGAC
cFos-R	AGTTGATCTGTCTCCGCTTGG
Icam1-F	GTGGGTCGAAGGTGGTTCTT
Icam1-R	CCAGCCGAGGACCATACAG
Itgad-F	CATTTCTGAGAGCCCAGGTGT
Itgad-R	GACGTGAGATGTGGAGGAGTC
Junb-F	CTTGATCGTCCCCAACAGCA
Junb-R	TGACAAAACCGTCCGCAAAG
Lcn2-F	TTCTCTGTCCCCACCGACCA
Lcn2-R	GGAAAGATGGAGTGGCAGACA
Lrg1-F	TGGGGACTATATAAAGCCACCTTC
Lrg1-R	CTTGAGATCCTGGAGGCTTCCTT
Lsp1-F	ACAGCAGACACTCATCAGCC
Lsp1-R	CAGATGCTCTTCCGCCTCC
Mmp8-F	CTTGCCCATGCCTTTCAACC
Mmp8-R	GGGGTTGTCTGAAGGTCCATA
Mmp9-F	CTCTGCTGCCCCTTACCAG
Mmp9-R	AGCGGTACAAGTATGCCTCTGC
Npas4-F	CTCTGGATGCTGATCGCCTT
Npas4-R	CAGGTGGGTGAGCATGGAAT
Nr4a1-F	GCGAAAGTTGGGGGAGTGT
Nr4a1-R	GCTTGAATACAGGGCATCTCCAG
Sctr-F	GTCTGGTCGGATTGGGTAGG
Sctr-R	GGCAGAGCTCCAACAGGGT
Sele-F	AAGCAAAGAAATTTGTTCCTGCTA
Sele-R	ACGATGCATTTGTGTTCCTGATT

Tlr6-F	AGAAGGAAGTCTTGAGCTTGGA
Tlr6-R	TATTAAGGCCAGGGCGCAAA
Nr4a1-F	GCGAAAGTTGGGGGAGTGT
Nr4a1-R	GCTTGAATACAGGGCATCTCCAG
Cdk2-F	TCCGGCTCGACACTGAGA
Cdk2-R	GGAAGAGGAATGCCCGTGAG
Cdk4-F	TTAGCCGAGCGTAAGGCTG
Cdk4-R	CCAGGCCGCTTAGAAACTGA
Cyclin D1-F	CAAAATGCCAGAGGCGGATG
Cyclin D1-R	CCAGGGCCTTGACCGGG
Opa1-F	TTCTGAGGCCCTTCTCTTGT
Opa1-R	TTCTTTGTCTGACACCTTCCTGT
Mfn1-F	CAGGGACGGAGTGAGTGTCC
Mfn1-R	TCTGGATTCCTGTATGTTGCTTCA
Cas3-F	GCTTGGAACGGTACGCTAA
Cas3-R	TCCGTACCAGAGCGAGATGA
Bcl2-F	CGTCGTGACTTCGCAGAGAT
Bcl2-R	TAGTTCCACAAAGGCATCCCAG
S100a9-F	GGAAGGAAGGACACCCTGAC
S100a9-R	GGCTTCATTTCTCTTCTCTTTCTTC
Gria2-F	ACCAATGCTTTCTGCTCCCA
Gria2-R	AGCAGAATCCAGCACAGCTT
Gria3-F	CCATGCTCTTGTCAGCTTCG
Gria3-R	AGTCCACCTATGCTGATGGT
Gria4-F	TACGACAAAGGAGAATGTGGCA
Gria4-R	GGCTTCGGAAAAAGTCAGCTTCAT
NMDA1-F	TCCTATGACAACAAGCGCGG
NMDA1-R	TGAAGTCCGATGATGCCGTC
BDNF-F	AGCGTCTTTTCCGAGGTTCG
BDNF-R	GGTGGAACTTCTTTGCGGCTT
PSD95-F	AGCCCCAGGATATGTGAACG
PSD95-R	GATGCTGTCGTTGACCCTGA
Mag-F	GGAGCCCAAGGGACTGTAAG
Mag-R	AGTGGCCTTTCAACCAAGTCT
Mog-F	TTACATGGAGGTTGGGCTTG
Mog-R	TATCACTCTGAATTGTCCTGCAT
Mbp-F	TTCACGACCCCGGAACATAG
Mbp-R	GGGTGTTCAAGAGTGGTGCT
Mobp-F	CCAGAAGACTAGCCGCCG
Mobp-R	CATCAGAGGGGACTTTGGCT
Plp1-F	CTGGCTGAGGGCTTCTACAC
Plp1-R	GTGATGCCCACAAACGTTGC
